# USP15 regulates SMURF2 kinetics through C-lobe mediated deubiquitination

**DOI:** 10.1038/srep14733

**Published:** 2015-10-05

**Authors:** Prasanna Vasudevan Iyengar, Patrick Jaynes, Laura Rodon, Dilraj Lama, Kai Pong Law, Yoon Pin Lim, Chandra Verma, Joan Seoane, Pieter Johan Adam Eichhorn

**Affiliations:** 1 Cancer Science Institute of Singapore, National University of Singapore, 117599, Singapore; 2 Vall d’Hebron Institute of Oncology (VHIO), Vall d’Hebron University Hospital, 08035 Barcelona, Spain; 3 Bioinformatics Institute (A*STAR), 30 Biopolis Street, 07-01 Matrix, 138671, Singapore; 4 Deparment of Biochemistry, Yong Loo Lin School of Medicine, National University of Singapore, Singapore; 5 Department of Biological Sciences, National University of Singapore, 117543, Singapore; 6 School of Biological Sciences, Nanyang Technological University, 60 Nanyang Drive, 637551, Singapore; 7 Department of Pharmacology, Yong Loo Lin School of Medicine, National University of Singapore, 117597, Singapore

## Abstract

Ubiquitin modification of the TGF-β pathway components is emerging as a key mechanism of TGF-β pathway regulation. To limit TGF-β responses, TGF-β signaling is regulated through a negative feedback loop whereby the E3 ligase SMURF2 targets the TGF-β receptor (TβR) complex for ubiquitin-mediated degradation. Counteracting this process, a number of deubiquitinating (DUBs) enzymes have recently been identified that deubiquitinate and stabilize the TβR. However the precise mechanism by which these DUBs act on TβR function remains poorly defined. Here, we demonstrate that apart from targeting the TβR complex directly, USP15 also deubiquitinates SMURF2 resulting in enhanced TβR stability and downstream pathway activation. Through proteomic analysis, we show that USP15 modulates the ubiquitination of Lys734, a residue required for SMURF2 catalytic activity. Our results show that SMURF2 is a critical target of USP15 in the TGF-β pathway and may also explain how USP15 and SMURF2 target multiple complementary protein complexes in other pathways.

Protein modification by ubiquitination is one of the key posttranslational modifications that regulate protein function. Ubiquitylation of substrates occurs through a ubiquitin (Ub) conjugation cascade involving the ubiquitin activating enzyme (E1), ubiquitin conjugating enzyme (E2), and the ubiquitin ligase enzyme (E3). The E3’s primary function is to facilitate the transfer of ubiquitin to the target substrate. The E3 ubiquitin ligases are primarily classified on the basis of their functional (catalytic) domain; the two major types are Really Interesting New Gene (RING) and the Homologues to E6-AP C Terminus (HECT). In the case of RING E3 ligases, the E3 acts as a scaffold bringing into proximity the substrate and the E1-activated E2-ubiquitin intermediate, permitting transfer of ubiquitin from the E2 directly to the substrate^1^. In contrast, with HECT E3 ligases, ubiquitin charged E2 transfers ubiquitin directly to the active site cysteine in its HECT domain via a transthiolation reaction^2^. Target lysine residues of the substrate subsequently attack the thioester bond which results in ubiquitin being transferred from the E3 to the substrate^2^.

The TGF-β family members, which include TGF-βs, Nodal and Bone Morphogenetic Proteins (BMPs) are secreted cytokines that regulate a vast array of biological activities. TGF-β ligand activation of the pathway is transmitted through the heterotrimeric complexes of type II (TβRII) and type I (TβRI) transmembrane Ser/Thr kinases receptors. Activated receptors initiate an intracellular signaling through phosphorylation of specific receptor regulated (R)-SMADs (SMAD2/SMAD3) in their C-terminal SXS motif 3. The phosphorylation of R-SMADs in their terminus creates an interaction interface that permits them to oligomerize with the co-SMAD, SMAD4^4^. Once formed, the SMAD complex translocates to the nucleus where they interact with various DNA binding proteins to regulate gene expression in a cell type and context specific manner.

Control of the desired TGF-β responses is tightly regulated through a number of different inhibitory mechanisms including ubiquitination. As part of a negative feedback loop, the inhibitor adaptor protein SMAD7, itself a direct transcriptional target of the SMAD complexes, acts as scaffold to recruit SMURF2, an E3 ubiquitin ligase of the C2-WW-HECT domain class, and the E2 ligase UBCH7 to the TGF-β receptor complex to facilitate receptor degradation and attenuate TGF-β signaling^5^,^6^. The binding of SMAD7 to SMURF2 also serves another purpose, SMURF2 has the ability to undergo autoubiquitination and therefore to maintain its stability and constrain unwanted activity towards its substrates, the C2 and HECT domains remain in a tightly closed conformation. The binding of SMAD7 to the HECT domain of SMURF2 abrogates the inhibitory intramolecular interactions between these domains, facilitating SMURF2 ubiquitin ligase activity^7^. Furthermore, the binding of SMAD7 to the activated receptor complex prevents access of R-SMADs to the receptor complex.

Ubiquitination is a reversible process, and ubiquitin moieties can be removed from polypeptides by deubiquitinating enzymes (DUBs). Recently, a number of DUBs have been identified that oppose the ubiquitination and degradation of the TGF-β receptor complex^8^,^9^,^10^,^11^. Therefore to better understand the cross functionality of these DUBs we sought to delineate the mechanism of how these DUBs affect TβR stability. Using a functional genetic screen we have previously found that USP15 forms a complex with SMAD7 and SMURF2 and is recruited to the TGF-β receptor complex, where it deubiquitinates and stabilizes TβRI^9^. In this scenario SMAD7 acts as an adaptor protein, interacting with two enzymes with opposing activities resulting in a constant balancing act between the ligase and DUB in regulating TGF-β output. Similarly, Peter ten Dijke and colleagues have demonstrated that TRAF4 facilitates the recruitment of USP15 to the TβR complex antagonizing SMURF2 mediated degradation of the complex^12^. We now establish that USP15 also directly deubiquitinates the E3 ligase SMURF2, opposing the activity of SMURF2 towards the TβR complex. Biochemically we demonstrate that USP15 regulates ubiquitination of a number of lysine residues on SMURF2 including a key residue in the C-Lobe of SMURF2 which has previously been suggested to be required for ubiquitin transfer from the E2 to the catalytic cysteine in SMURF2^13^.

## Results

### USP15 mediated activation of the TGF-β pathway is inhibited by a catalytically inactive mutant of SMURF2

**Figure 1:**
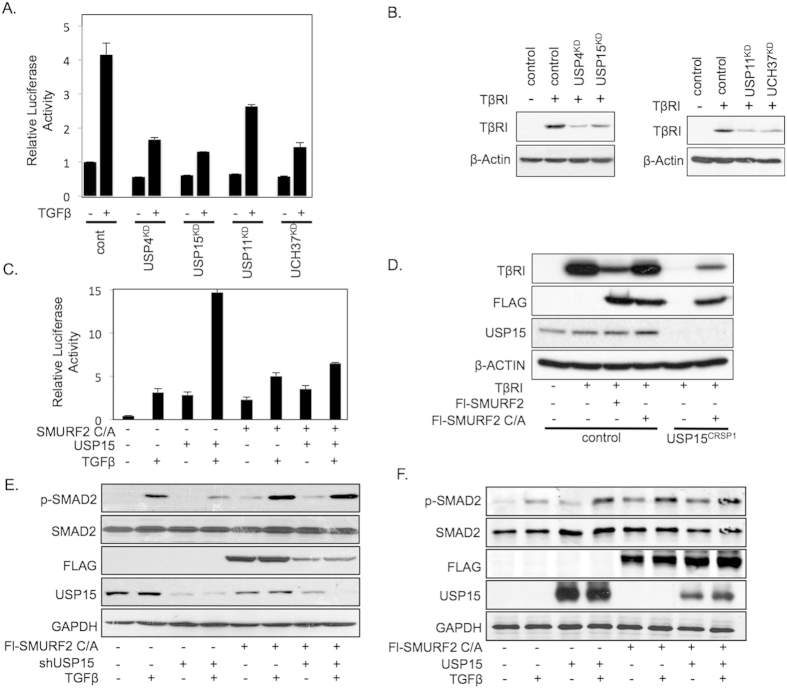
USP15 function in the TGF-β pathway is dependent on SMURF2 in HEK293T cells. (**A**) Luciferase assay in HEK293T cells transfected with CAGA-luciferase reporter and shRNAs targeting deubiquitinating enzymes USP4, USP11, USP15, or UCH37 treated with TGF-β (2.5 ng/ml). Data are mean ± s.d. (**B**) Immunoblot analysis showing levels of transfected TβRI along with shRNA vectors described in A. (**C**) Luciferase assay representing TGF-β activity in 293T cells transfected with CAGA-luciferase reporter and either catalytically inactive mutant of SMURF2 C/A, USP15, or both treated with TGF-β (2.5 ng/ml). Data are mean ± s.d. (**D**) Immunoblot analysis in 293T or USP15^CRSP1^ cells expressing TβRI and Flag-tagged SMURF2 or SMURF2 C/A. Whole cell lysates were probed with the indicated antibodies. (**E**) Immunoblot analysis with HEK293T cells showing changes in p-SMAD2 levels when transfected with shRNA targeting USP15, SMURF2 C/A or both and treated with TGF-β overnight (2.5 ng/ml). (**F)** Immunoblot analysis showing changes in p-SMAD2 levels when transfected with USP15, SMURF2 (C/A) or both and treated with TGF-β overnight (2.5 ng/ml).

The DUBs USP4, USP11, USP15, and UCH37 have previously been demonstrated to modulate TGF-β pathway activity by directly deubiquitinating the TβRI, resulting in increased TβRI stability (Fig. 1A,B)^8^,^9^,^10^,^11^. As DUBs USP11, USP15, and UCH37 all form a complex with SMAD7, we first assessed if DUB function is dependent on the ubiquitination of TβR-I by SMURF2, the E3 ligase, which binds to SMAD7 and targets the TβR complex for degradation. We co-transfected HEK293T cells with the TGF-β-responsive luciferase reporter (CAGA-Luc) and each of the individual DUBs with a catalytically inactive mutant of SMURF2 (Cys716Ala, C/A). Consistent with previous reports, overexpression of USP4, 11, and 15 significantly enhanced the activity of the reporter (Fig. 1C; Sup. Fig. 1A, B). In contrast, UCH37 demonstrated a minimal increase in CAGA-Luc activity suggesting that UCH37 may also target the TGF-β pathway downstream of the TβR complex to regulate overall TGF-β signaling (Sup. Fig. 1C). Surprisingly, co-expression of catalytically inactive SMURF2 completely abolished the ability of USP15 to activate this reporter (Fig. 1C). In contrast, USP4 activity was unaffected by the co-expression of SMURF2 C/A while USP11 activity was only partially inhibited (Sup. Fig. 1A, B). Thus, USP15 regulation of the TGF-β pathway is in part dependent upon the function of SMURF2. We therefore decided to focus our attention on USP15.

To investigate the role of USP15 in the TGF-β pathway we generated null alleles for USP15 in 293T cells using the type II CRISPR (Clustered Regularly Interspaced Short Palindromic Repeats) system. Guide RNA was cloned into the PX330 vector which permits both expression of guide RNA and Cas9 on a single expression plasmid. 293T cells were transfected and a single independent clone was isolated that carried frameshift mutations on both alleles resulting in a USP15 null expression cell line, USP15^CRSP1^ (Sup. Fig. 2A, B). USP15^CRSP1^ cells exhibited a significant decrease in TβR-I stability resulting in overall downregulation of the TGF-β pathway as determined by a decrease in activity of the TGF-β responsive reporter, CAGA-Luc (Sup. Fig. 2C, D). The loss of TβRI stability in USP15 targeted CRISPR cell lines was reverted by complementation with ectopically expressed USP15, indicating that the observed phenotype is due to loss of USP15 expression and not through unwanted off target effects (Sup. Fig. 2C). Next, we analysed whether degradation of TβRI in USP15 null cells was attenuated in the presence of catalytically inactive SMURF2. Indeed, the inhibitory effect of USP15 knockout was diminished upon co-expression of SMURF2 C/A (Fig. 1D). To further examine if USP15 activity in the TGF-β pathway is dependent upon SMURF2 function we generated CRISPR/CAS knockout cells for SMURF2 (Sup. Fig. 3A, B). As expected, SMURF2^CRSP1^ cells displayed enhanced TβRI stability and overall activation of the TGF-β pathway, which was dependent on SMURF2 as reintroduction of SMURF2 inhibited these effects (Sup. Fig. 3C, D, E, F, G). Importantly, co-expression of USP15 annulled the inhibitory actions of SMURF2 on the TGF-β pathway (Sup. Fig. 3F, G). In contrast, ectopic expression of USP15 did not augment TβRI levels or overall TGF-β luciferase activity in SMURF2^CRSP1^ cell lines when expressed alone or in combination with SMURF2 C/A indicating that USP15’s role in the TGF-β pathway is dependent on SMURF2 function (Sup. Fig. 3F, G). Similar effects were observed when we ectopically expressed SMURF2 C/A (Sup. Fig. 3G). It has been described that a catalytically inactive version of SMURF2 can act as a dominant negative through sequestration of TβRI, preventing endogenous SMURF2 interaction, resulting in overall TβR stability. However, we do not observe this effect in luciferase reporter assays indicating that in this context, the effect of SMURF2 C/A on endogenous SMURF2 is negligible.

We then assessed the relationship of these proteins on the downstream activation of R-SMADs. In line with previous results, TGF-β induced phosphorylated SMAD2 (p-SMAD2) was decreased upon suppression of endogenous USP15. However, the inhibitory effect of USP15 knockdown was again attenuated upon co-expression of SMURF2 C/A (Fig. 1E). Similarly, ectopic expression of USP15 and SMURF2 C/A did not enhance p-SMAD2 levels compared with either construct alone (Fig. 1F). Taken together, these results suggest that USP15 may function through SMURF2 to regulate TβRI stability and downstream TGF-β signals.

### USP15 regulates the ubiquitination of the E3 ligase SMURF2

**Figure 2:**
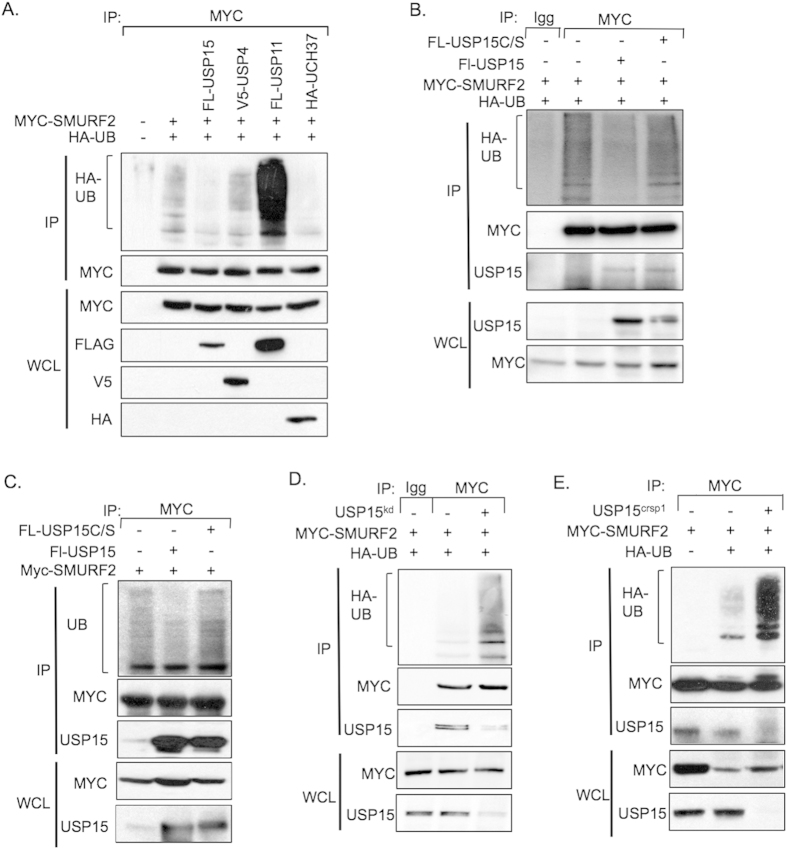
USP15 targets SMURF2 for deubiquitination. (**A**) Immunoprecipitation with anti-Myc antibodies in HEK293T cells overexpressing Myc-SMURF2, the indicated deubiquitinating enzymes and hemagglutinin (HA)-tagged ubiquitin (HA-UB). Immunoblot analysis probed for the indicated proteins is shown. (**B**) Immunoprecipitation analysis with 293T cells expressing SMURF2, USP15 or USP15 C/S and HA-UB; an immunoblot analysis of the indicated proteins is shown. (**C**) Immunoprecipitation analysis with 293T cells expressing SMURF2 and USP15 or USP15 C/S; an immunoblot analysis of the indicated proteins is shown. (**D**) Immunoprecipitation in HEK293T transfected with Myc-SMURF2, HA-ubiquitin and an shRNA against USP15, cells were immunoprecipitated with either mouse IgG or Myc antibodies; immunoblot analysis of the indicated proteins is shown. (**E**) Immunoprecipitation with anti-Myc antibodies using control or USP15^CRISPR^ cell lines, overexpressing Myc-SMURF2 and HA-ubiquitin.

In light of the data presented so far we reasoned that USP15 may also directly deubiquitinate SMURF2. To reveal ubiquitinated isoforms of SMURF2 we co-transfected HEK293T cells with expression plasmids encoding SMURF2, HA-tagged ubiquitin, and either USP15, USP4, USP11, or UCH37. Interestingly, all three DUBs that form a complex with SMAD7 and therefore potentially SMURF2 affected the ubiquitination levels of SMURF2. Both USP15 and UCH37 deubiquitinated SMURF2 to varying degrees, however, the effect of USP15 on SMURF2 was consistently more robust (Fig. 2A). USP11 enhanced SMURF2 ubiquitination, indicating that the effect of USP11 on SMURF2 function is indirect (Fig. 2A). Overexpression of USP4 did not alter the ubiquitination status of SMURF2. Furthermore, wild-type USP15, but not a catalytically inactive version of USP15, USP15 C269S (USP15 C/S), caused a marked reduction in SMURF2 ubiquitination (Fig. 2B). Similar results were observed when we analyzed endogenous ubiquitination levels of SMURF2 in the presence of USP15 or USP15 C/S (Fig. 2C).

To see if USP15 deubiquitinated SMURF2 *in vitro*, we purified USP15 and Ub-SMURF2 from extracts of HEK293T cells and proteins were mixed under conditions supporting SMURF2 deubiquitination. As seen in supplementary Fig. 4A, USP15 but not USP15 C/S reduced the levels of Ub-SMURF2. Furthermore, *in vitro* deubiquitination of SMURF2 was unique to USP15 since the co-administration of other DUBs to Ub-SMURF2 did not significantly decrease overall SMURF2 ubiquitination levels (Sup. Fig. 4B).

Consistent with these results, suppression of USP15 significantly increased the incorporation of ubiquitin into SMURF2 in both the endogenous setting and in cell lines ectopically expressing HA-Ub (Fig. 2D, Sup. Figure 4C). To test if USP15 null cells enhanced the ubiquitination of SMURF2, we co-transfected plasmids expressing SMURF2 and HA-tagged ubiquitin into 293T cells or their USP15 null counterparts. USP15 knockout cells exhibited a robust increase in the ubiquitination levels of SMURF2 compared to wild type 293T cells (Fig. 2E). These data demonstrate that USP15 regulates SMURF2 ubiquitination *in vivo* and *in vitro*. Furthermore, it is important to note that ectopic expression of USP15 did not dramatically alter steady state levels of SMURF2 suggesting that USP15 regulates SMURF2 function through other mechanisms independent of proteasome degradation.

### Characterization of ubiquitinated lysine residues on SMURF2

**Figure 3:**
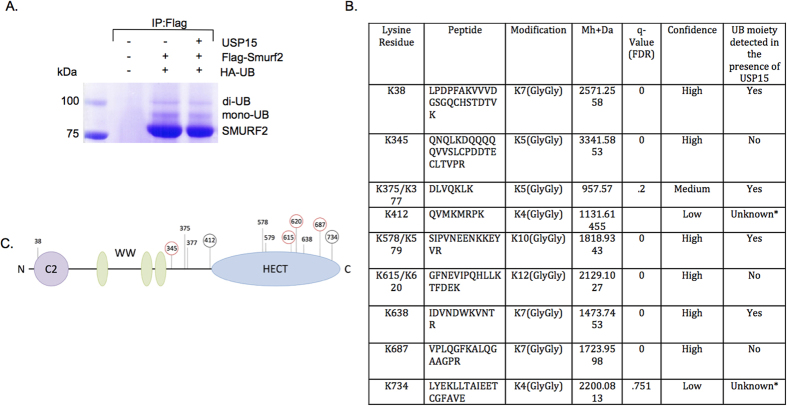
USP15 deubiquitinates multiple lysine residues on SMURF2. (**A**) Immunoprecipitation using anti-Flag resin in HEK293T cells, transfected with control vector (lane 1) or Flag-SMURF2 (lanes 2 and 3), K0-ubiquitin, and SMAD7 with or without USP15 overexpression and visualized using coomassie staining; bands above Flag-SMURF2 show mono and di-mono-ubiquitinated Flag-SMURF2. (**B**) Table showing candidate ubiquitinated lysine residues derived from mass spectrometric analysis. *Indicates non-detectable peptides by MASS SPEC. (**C**) Schematic representation of SMURF2 with its domains and candidate ubiquitination sites; ubiquitinated sites not detected (red circle) or detected with low confidence (black circle) in the presence of USP15 are indicated.

Ubiquitination of substrates occurs primarily through the binding of the Gly76 of Ub to a Lys (K) residue on the substrate resulting in the formation of an isopeptide bond between the carboxylic acid group of Gly76 and the epsilon amino group of the target lysine. However, recent data has demonstrated that non-covalent ubiquitin binding to the HECT domain of E3 ligases may play a role in regulating polyubiquitination^14^,^15^. Nevertheless, most DUBs, including the USP family of DUBs, specifically target isopeptide bond formation. Therefore to identify potential Ub residues targeted by USP15 on SMURF2 we performed mass spectrometry specifically mapping ubiquitinated lysines. Samples were prepared by transfecting HEK293T cells with Flag-tagged SMURF2 and HA-tagged Ub with or without USP15. SMURF2 was subsequently immunoprecipitated with FLAG resin beads and protein samples were separated by SDS-PAGE (Fig. 3A). Mono-ubiquitinated and multi-mono-ubiquitinated isoforms of SMURF2 were isolated, digested with trypsin and prepared for analyses. We identified 12 novel ubiquitination sites with a greater than 80% coverage of lysine residues in SMURF2 (Fig. 3B,C, Sup. Figure 5 and data not shown). Cross-analyses of ubiquitination predictor programs identified a number of complementary putative ubiquitination sites including K38, K345, K375, K377, K687, and K734 (http://www.ubpred.org, http://protein.cau.edu.cn/cksaap_ubsite/, and http://bdmpub.biocuckoo.org/results.php). Importantly, several of the ubiquitinated residues appear to be regulated by USP15 as overexpression of USP15 decreased the levels of a number of ubiquitinated peptides including, K615, K620, and K687 (Fig. 3B,C). Of note, low confidence spectra data of ubiquitinated peptides K412 and K734 were the result of a miscleavage event (Fig. 3B). Both loci contain multiple Arg and Lys residues (susceptible to tryptic digestion) rendering the resulting digested peptides too small to be detected by standard MASS SPEC. These results indicate that SMURF2 forms covalent isopeptide bonds with ubiquitin at multiple residues along its external surface, a subset of which can be targeted for deubiquitination by USP15.

**Figure 4:**
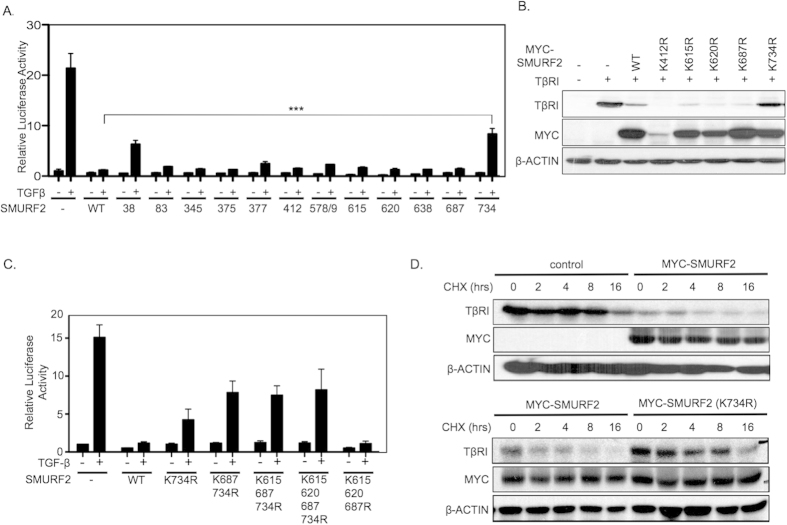
Lysine 734 of SMURF2 is critical for SMURF2 function. (**A**) Luciferase assay showing relative TGF-β activity in HEK293T cells transfected with CAGA-Luc and either wild-type SMURF2 or SMURF2 mutants representing candidate ubiquitin sites and treated with TGF-β overnight (2.5 ng/ml). ***P value = 0.0002 using Student’s *t* test. Data are mean ± s.d. (**B**) Immunoblot analysis in HEK293T cells expressing TβRI in the presence of either wild-type SMURF2 or SMURF2 mutants as described in A. (**C**) Luciferase assay in HEK293T cells transfected with CAGA-luciferase reporter with wild-type SMURF2, SMURF2 K734R or in combination with other candidate deubiquitination sites of the HECT domain of SMURF2 and treated with TGF-β overnight (2.5 ng/ml). Data are mean ± s.d. (**D**) HEK293T cells were transfected with TβRI and either control, Myc-SMURF2 or Myc-SMURF2 K734R; the cells were treated with cycloheximide (10 μg/ml) and TGF-β (2.5 ng/ml) and lysed at the indicated time points.

To further understand the functional significance of the potential Ub sites we prepared a series of SMURF2 mutants harboring lysine to arginine substitutions and tested SMURF2 activity by its ability to inhibit TGF-β induced CAGA-luciferase activity. Although most SMURF2 mutants behaved similar to the wild type counterpart we found that mutations at Lys38 (K38R) and Lys734 (K734R) significantly decreased SMURF2 activity (Fig. 4A). Both USP15 and SMURF2 target multiple components of the TGF-β pathway therefore we sought to provide experimental evidence that the candidate USP15 deubiquitination sites on SMURF2 had the ability to rescue TβRI stability^9^,^16^,^17^. To this end, we co-transfected HEK293T cells with TβRI and SMURF2 or its corresponding mutants. We observed that only mutations at Lys734 caused a pronounced attenuation of SMURF2 mediated degradation of TGF-β receptor levels (Fig. 4B, Sup. Fig. 6A). Since our results and those of others indicate that SMURF2 may undergo various ubiquitination events (mono, multi-mono, or polyubiquitination), we reasoned that ubiquitination at multiple residues may have a cumulative effect on SMURF2 activity^7^. We took into focus the candidate USP15 deubiquitination sites on the HECT domain of SMURF2 (K615, K620, K687) that reside in close proximity to Lys734. We found that the combined mutation of Lys687 and Lys734 partially enhanced the rescue of TGF-β activity compared to Lys734 mutant alone (Fig. 4C). However, mutation of Lys615 and Lys620 did not further enhance this effect. Furthermore, Lys734 was absolutely crucial to this effect as a SMURF2 variant mutated only at Lys615, Lys620, and Lys687 completely inhibited luciferase activity in line with wild-type SMURF2 (Fig. 4C). Next, we assessed the impact of Lys734 mutation on TβRI stability over time. We followed the degradation pattern of TβRI in HEK293T cells transfected with either wild type SMURF2 or SMURF2 K734R and exposed to TGF-β and cyclohexamide. Ectopic expression of SMURF2 increased receptor degradation over time with respect to control cells (Fig. 4D, Sup. Fig. 6B). In contrast, in SMURF2 K734R expressing cells, TβRI degradation was dramatically reduced (Fig. 4D, Sup. Figure 6C). Furthermore, ectopic expression of SMURF2 K734R completely attenuated SMURF2 inhibition of phosphorylated SMAD2 following the addition of TGF-β (Sup. Fig. 6D). Taken together our results indicate that Lys734 is a critical residue on SMURF2 that is required for effective targeting of the TβRI for degradation and overall downregulation of TGF-β activity.

### Lysine 734 of SMURF2 is critical for the function of USP15 in the TGF-β pathway

**Figure 5:**
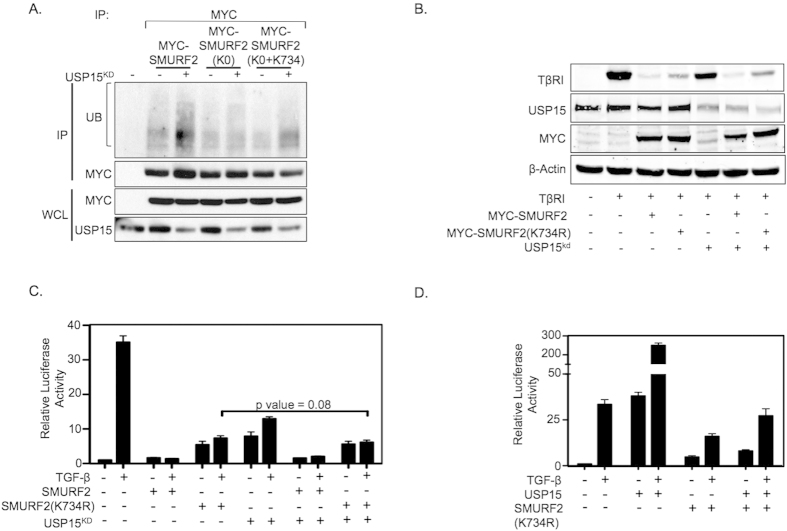
USP15 exerts its effect on SMURF2 through lysine 734. (**A**) Immunoprecipitation analysis with anti-Myc antibodies in HEK293T cells expressing either wild-type Myc-SMURF2 or Myc-SMURF2 with all the candidate deubiquitination sites mutated including or excluding lysine 734, indicated by K0 or K0-K734, respectively. (**B**) Immunoblot analysis using HEK293T cells transfected with the indicated plasmids, β-actin is shown as loading control. (**C**) Luciferase assay representing TGF-β activity in HEK293T cells transfected with the indicated plasmids either alone or together with knockdown vectors targeting USP15 and treated with TGF-β overnight (2.5 ng/ml). *P* value = 0.08 using Student’s *t* test. Data are mean ± s.d. (**D**) Luciferase assay representing TGF-β activity in HEK293T cells transfected with the indicated plasmids either alone or together with ectopic expression of USP15 and treated with TGF-β overnight (2.5 ng/ml). Data are mean ± s.d.

Autoubiquitination of SMURF2 family members (SMURF1/2-NEDD4-Rsp5P) have been determined to be a critical factor in their ability to ubiquitinate their respective substrates^18^. Our results indicate that USP15 directly deubiquitinates SMURF2 potentially resulting in the inhibition of SMURF2 catalytic activity. As Lys734 appears to be a critical residue for SMURF2 activity in the TGF-β pathway we sought to investigate if USP15 regulates Lys734 ubiquitination. To specifically reveal Lys734 ubiquitinated isoforms of SMURF2 we generated a SMURF2 variant mutated at all ubiquitination sites identified by MASS SPEC, which are potentially regulated by USP15, SMURF2 (K0), and a SMURF2 mutant wild type only at Lys734 (K0-K734). Importantly, when we analyzed the ubiquitination pattern of these SMURF2 mutants in lysates of transfected HEK293T cells in the presence of USP15 knockdown vectors, we found that, in addition to wild type SMURF2, loss of USP15 only enhanced SMURF2 ubiquitination in the SMURF2 (K0–734) variant, but not in SMURF2 (K0) “lysine less” mutant (Fig. 5A). These results suggest that Lys734 of SMURF2 is not only ubiquitinated but also a target site for USP15.

Since USP15 can potentially regulate SMURF2 ubiquitination at Lys734 we examined whether USP15 is able to regulate TβRI stability in the presence of a SMURF2 K734 mutant. To this end we co-transfected HEK293T cells with SMURF2 K734 in the presence or absence of knockdown vectors targeting USP15. In line with previous results, Lys734 partially attenuated SMURF2 mediated degradation of TβRI. Strikingly, whereas TβRI stability was sensitive to USP15 knockdown, cells co-transfected with SMURF2 K734 were completely insensitive to USP15 depletion (Fig. 5B). Furthermore, luciferase assay results demonstrate that overall TGF-β activity in the presence of over-expressed SMURF2 K734 remains relatively unchanged regardless of the expression levels of USP15 (Fig. 5C,D). Taken together, these results suggest that ubiquitination at Lys734 of SMURF2 is a required determinant for USP15 function in the TGF-β pathway.

### SMURF2 requires lysine 734 for transthiolation

**Figure 6:**
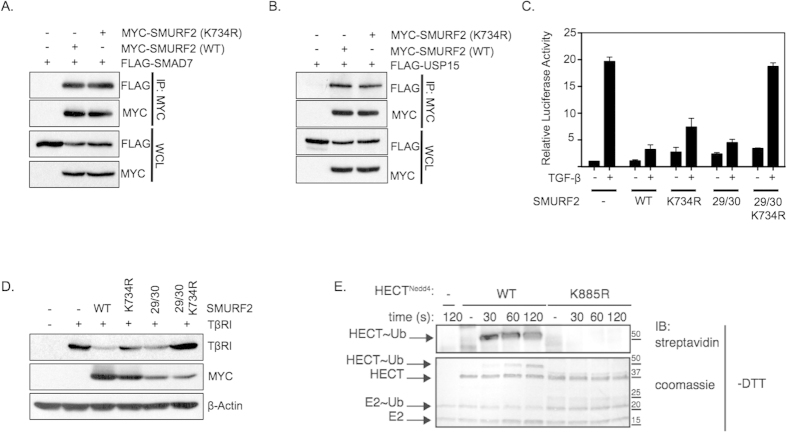
Lysine 734 of SMURF2 is required for SMURF2 transthiolation. (**A**) Immunoprecipitation with anti-Myc antibodies in HEK293T cells transfected with Flag-SMAD7 and either with Myc-SMURF2 or Myc-SMURF2 (K734R); whole cell lysates are indicated below (WCL). (**B**) Immunoprecipitation with anti-Myc antibodies in HEK293T cells transfected with Flag-USP15 and either with Myc-SMURF2 or Myc-SMURF2 (K734R). (**C**) Luciferase activity in HEK293T cells transfected with CAGA-luciferase, Myc-SMURF2, Myc-SMURF2 (K734R), Myc-SMURF2 (FF29/30AA) or Myc-SMURF2 (FF29/30AA+K734R) treated with TGF-β (2.5 ng/ml). Data are mean ± s.d. (**D**) Aforementioned plasmids were co-transfected with TβRI in 293T cells; an immunoblot analysis of the indicated proteins is shown. (**E**) An *in vitro* trans-thiolation assay; purified HECT domain of NEDD4 (either wild-type or K885R) was incubated with E1, E2 and ubiquitin for the indicated time points and analyzed through immunoblotting or coomassie staining.

Next, we investigated how mutating Lys734 would alter the molecular function of SMURF2. Work in recent years has demonstrated that SMAD7 acts as a key regulator of many HECT type E3 ubiquitin ligases in the TGF-β pathway primarily by optimizing the interaction surfaces of these proteins^6^,^7^,^19^. The binding of SMAD7 to SMURF2 functions to limit the intramolecular interactions between the C2 and HECT domains of SMURF2, essentially opening up the protein and permitting the recruitment of the E2 ligase to the HECT domain. Initially, we tested if mutated SMURF2 could form complexes with both SMAD7 and USP15. As shown in Fig. 6A,B, mutated SMURF2 bound to both USP15 and SMAD7 to the same degree as wild type SMURF2. We also investigated whether Lys734 played a role in modulating the intramolecular interactions between the C2 and HECT domains. To that end, we introduced a Lys734 mutation into an activated form of SMURF2 mutated at two residues (F29A/F30A), which are required to form the interaction interface between the C2 and HECT domains^7^. Under these conditions we speculated that if Lys734 is involved in abrogating the intramolecular interactions between the C2 and HECT domains, mutation of Lys734 would be incapable of inhibiting SMURF2 activity in the presence of F29A/F30A. Strikingly, mutation at Lys734 completely inhibited the active form of the SMURF2 (F29A/F30A) mutant to a greater degree then Lys734 mutation alone (Fig. 6C,D). This indicates that the role of ubiquitination at Lys734 on SMURF2 activity is independent of the autoinhibitory effect the C2 domain plays on HECT function.

As the C-lobe portion of the HECT domain has previously been implicated in anchoring the Ub from the E2 prior to thioester bond formation, it may suggest that Lys734 is required to prime SMURF2 for catalysis. To directly test this we performed a transthiolation assay on NEDD4, a SMURF2 family member containing the same conserved lysine residue within its C-Lobe. As shown in Fig. 6E mutation of Lys885 completely impaired the ability of the protein to receive Ub from the E2 resulting in the inability of Ub to form a thioester bond with NEDD4. Taken together, our results show that Lys734 may be a critical site for the enzymatic steps leading to the catalytic ubiquitination of SMURF2.

### Lys734 is required for SMURF2 inhibition of TGF-β biological effects

**Figure 7:**
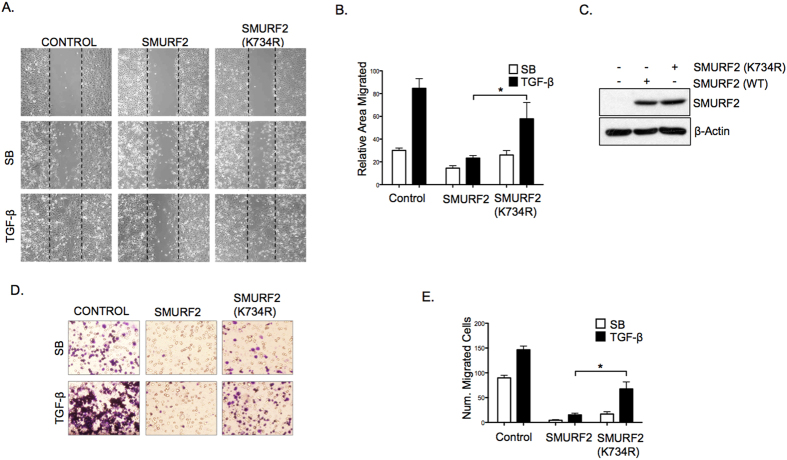
Mutating lysine 734 of SMURF2 mitigates TGF-β induced migration. (**A**) MDA-MB-231 cells stably expressing SMURF2 (wild-type) or SMURF2 (K734R) were plated for a scratch assay and treated with SB431542 (5 μM) or TGF-β (5 ng/ml), panels show migration at 0 and 24 hours. (**B**) Percentage of migrated area within the black dotted lines was estimated with respect to control (0 hour) and a graph was plotted. *P value = 0.014 using Student’s *t* test. Data are mean ± s.d. (**C**) Immunoblotting showing lentiviral induced SMURF2 (wild-type) or SMURF2 (K734R) expression in MDA-MB-231 cells. (**D**) Transwell assay of MDA-MB-231 cells infected with SMURF2 or SMURF2 (K734R) and treated with SB431542 (5 μM) or TGF-β (5 ng/ml) for 16 hours before fixation and staining with crystal violet. (**E**) Graph represents total number of migrated cells from four random fields from Figure D. *P value = 0.011 using Student’s *t* test. Data are mean ± s.d.

Depending on cellular context TGF-β can either elicit a potent cytostatic response or promote tumorigenesis through a number of biological responses, including cell migration. As SMURF2 is a potent inhibitor of TGF-β activity we investigated whether introducing a K734R mutant of SMURF2 into the TGF-β responsive metastatic breast cancer cell line MDA-MB-231 compromises the ability of SMURF2 to reduce TGF-β dependent migration. MDA-MB-231 cells were infected with either control vector, SMURF2 (wild-type) or SMURF2 (K734R) via lentiviral transduction (Fig. 7C) and migration was quantified by scratch assays and by transwell migration assays (Fig. 7A,B,D,E). In line with previous results, SMURF2 K734R partially abolished SMURF2 mediated inhibition of TGF-β induced migration. Taken together these results show that Lys734 is crucial for SMURF2 function in regulating TGF-β biological effects.

## Discussion

The SMURF2 E3 ligase regulates the fate of numerous proteins involved in cellular signaling therefore precise regulation of its activity is required. SMURF2 activity is stimulated by the binding of SMAD7, which abrogates the intramolecular interactions between the C2 and HECT domains permitting SMURF2 autoubiquitination and activation. SMAD7 also recruits SMURF2 to the TGF-β receptor complex to facilitate its ubiquitination. This leads to proteasome mediated degradation of the SMAD7/SMURF2/TβR complex and attenuation of TGF-β signal. Counteracting this process, a number of DUBs have been identified which deubiquitinate the TGF-β receptor resulting in its stabilization. The mechanistic details, however, of these events remain ambiguous. Here our results suggest that the deubiquitinating enzyme USP15 intricately controls SMURF2 ligase activity by regulating C-Lobe ubiquitination resulting in the loss of SMURF2 catalytic activity. The fact that USP15 simultaneously targets both the E3 ligase and its respective substrate, TGF-β receptor, is unsurprising. In a similar fashion the ubiquitin specific protease HAUSP also targets both the E3 ligase, MDM2, and its substrate p53^20^,^21^. Furthermore, recent reports have identified a number of shared substrates of SMURF2 and USP15 including MDM2, SMAD3, and RNF20, suggesting that the association between SMURF2 and USP15 may serve as a common regulatory method to control multiple analogous cellular protein complexes^16^,^22^,^23^,^24^,^25^,^26^.

During the ubiquitination transfer cascade, the C-Lobe of the HECT domain forms multiple non-covalent interactions with the E2 bound Ub, anchoring Ub in close proximity to the catalytic cysteine (C716) thereby allowing a thioester bond to form between the catalytic cysteine of SMURF2 and the incoming Ub^13^,^27^. One of these interacting residues in the C-lobe appears to be Lys940 of NEDD4L, which forms a hydrogen bond between the backbone oxygen of Gly35 of Ub^13^. Sequence analyses confirmed that NEDD4L Lys940 is a conserved residue in all of the NEDD4 family members including, NEDD4 (Lys885) and SMURF2 (Lys734) (Sup. Fig. 7). Previous reports have demonstrated that mutations affecting the interaction between the HECT domain and the incoming Ub disrupt the transfer of Ub from the E2 to the E3 and thioester bond formation^13^,^27^. Similarly, our data clearly indicates that residue Lys885 of NEDD4 is also absolutely required for the formation of the thioester bond at the catalytic cysteine (Fig. 6E). Following transthiolation the interaction between Lys885 and the catalytically bound Ub is not observed, potentially freeing up Lys885 for post-translational modifications^27^. Importantly, our data suggests that the corresponding residue on SMURF2 Lys734 may be ubiquitinated. However, isopeptide bond formation due to a resulting bound ubiquitin at Lys734 prior to E2 Ub transfer would impair potential hydrogen bond formation and limit Ub transfer from E2 to SMURF2, in essence creating a partial dominant negative version of SMURF2. Therefore, we hypothesize a two-step process whereby ubiquitination at Lys734 occurs following Ub transfer from the E2 to the E3.

**Figure 8:**
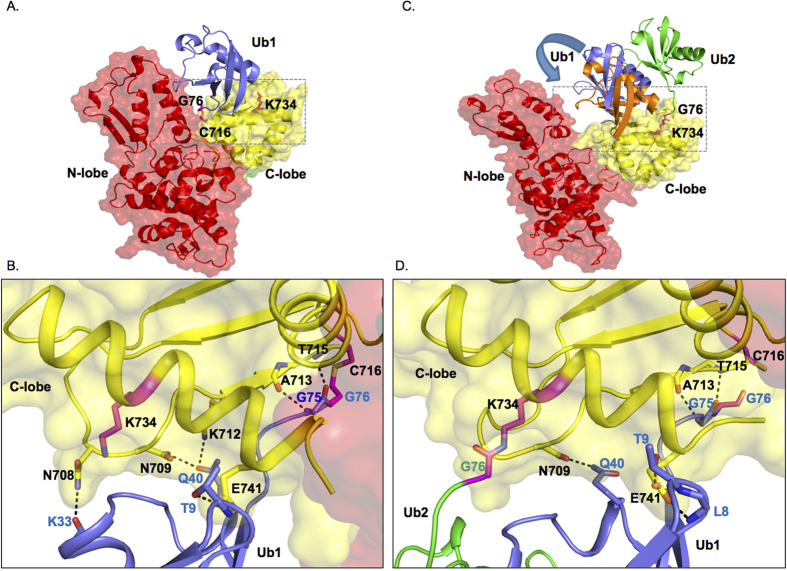
Model for Ub-loaded SMURF2 HECT domain in complex with Ub bound at lysine 734. (**A**) Molecular model of donor ubiquitin (Ub1, blue) bound to the catalytic pocket of HECT domain of SMURF2. (**B**) Inset of A showing the details of the hydrogen-bond interaction between SMURF2 C-Lobe (yellow) and catalytically bound Ub1 (blue). Thioester bond between Gly76 and Cys716 is denoted. Unbound Lys 734 is also shown. (**C**) Molecular model of ubiquitin (green, Ub2) bound to Lys734 site with donor ubiquitin (Ub1) bound to the catalytic pocket. The displacement of Ub1 (shown in blue and orange) due to the presence of Ub2 is indicated by an arrow. (**D**) Inset of C showing the hydrogen-bond interactions between SMURF2 C-Lobe (yellow) and Ub1 (blue) in the presence of Lys734 bound Ub2 (green). The isopeptide bond between Lys734 and Gly76 of Ub2 is also shown.

To better understand the essential dynamics underlying C-Lobe mediated ubiquitination we modeled the three dimensional structure of Ub-loaded SMURF2 structure and probed the associated dynamics by subjecting it to molecular dynamics (MD) simulations. Basic analysis of these simulations are described in supplementary figures S8A, S8B, S8C, and S8D. Our observations reveal that upon thioester bond formation Lys734 no longer forms a hydrogen bond with the catalytically bound Ub potentially permitting Lys734 to form an isopeptide bond with a secondary Ub (Fig. 8A,B). Next, we also tested the effect of Ub bound to Lys734 on the donor ubiquitin (Ub1). Our simulation showed that the bound state conformation of Ub1 with respect to the C-lobe domain of SMURF2 is influenced by Ub2 (Fig. 8C,D, Sup. Fig. 9A, Supplementary Movies S1 and S2). Quantification of the inter-helical angle between the pair of helices H1 and H2 present in Ub1 and C-lobe domain of SMURF2 respectively indicate a significant alteration in the angle in SMURF2:Ub1:Ub2 system (Sup. Fig. S9A and S9B). This measurement is a clear indication of the change in the orientation of Ub1 relative to the C-lobe domain in the presence of Ub2. To further characterize the effect of this change on Ub1, we have investigated the interactions between Ub1 and SMURF2. As indicated in Table S1 interactions between Ub1 and SMURF2 are a combination of both van der Waals and hydrogen-bond interactions. It is clear from the data, that despite some common set of residue-pairs that are in contact in both the simulations, we observe a distinct difference in the interactions that are formed between SMURF2:Ub1:Ub2 and SMURF2:Ub1 systems (Fig. 8B,D). Taken together, these data indicate that the conformational reorientation of Ub1 in the presence of Ub2 does indeed cause a change in its interaction profile with SMURF2 and hint that ubiquitination at Lys734 may be an important factor for the efficient transfer of Ub from the E3 to its substrate.

Following Ub ligation, USP15 may subsequently function to regulate Lys734 ubiquitination permitting the HECT domain to receive another Ub from the E2. Recent reports have indicated that the C-Lobe forms non-covalent interactions with donor Ub to determine ubiquitin chain specificity suggesting the possibility that Lys734 may also play a role in donor Ub availability^13^,^28^. Additional investigation will be required to fully understand how post-translational modifications of C-Lobe affects non-covalently bound Ub and the formation of polyubiquitin chains. An outstanding question remains as to the identity of the ligase that targets Lys734 for ubiquitination. It seems unlikely that Lys734 ubiquitination is a result of SMURF2 autoubiquitination as this would counteract any further E2-E3 Ub transfer. TRAF4 has recently been shown to ubiquitinate SMURF2, but TRAF4 also seems like an unlikely candidate since TRAF4 ubiquitination targets SMURF2 for proteosomal degradation. Further experimentation will be required to elucidate the mechanism behind Lys734 ubiquitination.

One of the more interesting findings of our study is the difference in SMURF2 activity when Lys734 is mutated in wild type SMURF2 compared to when it is mutated in the SMURF2 (F29A/F30A) variant (Fig. 6C). There are several potential implications of these observations. One such implication is that upon the binding of SMAD7 and the loss of the intramolecular interactions between the N-Lobe and C-Lobe, overall SMURF2 activity in the TGF-β pathway is absolutely dependent upon Lys734 integrity for appropriate E2 to E3 ubiquitin transfer. In contrast, mutation of Lys734 in wild type SMURF2 only partially inhibited SMURF2 activity. (Figs 4A,C,5A and 6C). As our data indicates, Lys734 is critical for transthiolation and activation of SMURF2, thus suggesting that SMURF2 may play an inhibitory function in TGF-β signaling independent of substrate ubiquitination. Conversely, transfer of Ub in Lys734 mutants from the E2 to E3 may still be occurring albeit at a lower kinetic rate undetectable in our experiments, permitting low levels of SMURF2 activity.

Our findings accentuate the complexity of the HECT ligase ubiquitination cascade but they also reveal a weakness that may be exploited in order to effectively target these enzymes. SMURF2 is frequently amplified in a number of solid tumors however, effective targeting of HECT domain ligases remains elusive (cBioPortal). Recent data has demonstrated that inhibition of SMURF2 by genetic means enhances MEK inhibitor sensitivity in melanoma^29^. One implication of our work may be that specific targeting of the C-Lobe of SMURF2 may potentially be utilized as a successful strategy to down regulate SMURF2 activity in a context dependent manner.

## Materials and Methods

### Cell culture, transfection and immunoblotting

HEK293T cells were maintained in standard Dulbecco’s modified Eagle’s medium (DMEM, Gibco) supplemented with 4.5 g/L D-Glucose, L-Glutamine and 110 mg/L Sodium Pyruvate. The media was further supplemented with 10% Fetal Bovine Serum (Gibco, Origin: United States) and 1% antibiotics (penicillin/streptomycin, Gibco). Cells were grown in a 5% CO2 atmosphere at 37 °C. For MDA-MB-231 cells, the media was further supplemented with 1% non-essential amino acids (Gibco). Transfection was done using calcium chloride and HEPES buffered saline (pH 6.95). Sixteen hours post-transfection, media was aspirated and cells were washed twice with 1x PBS and replenished with fresh media. 24 hours later, cells were washed twice with 1x PBS and lysed with RIPA lysis buffer (50 mM Tris-HCl pH 7.4, 150 mM NaCl, 1% Nonidet P-40, 0.1% SDS and 0.5% sodium deoxycholate) supplemented with Protease inhibitor cocktail (Complete EDTA-free tablet, Roche) and phosphatase inhibitors (50 mM sodium fluoride, 10 mM β-glycerophosphate and 1 mM sodium orthovanadate). The cells were lysed on ice before spinning down at 12,000 rpm for 15 minutes, supernatants were transferred to new tubes and protein estimation was done using BCA Protein estimation kit by ThermoScientific; 30 μg of lysates were boiled in sample buffer containing 10% β-mercaptoethanol and loaded onto a 10% Acrylamide SDS-PAGE gel. Immunoblotting was done on a PVDF membrane using wet transfer method. The membrane was blocked with non-fat skimmed milk for an hour at room temperature before probing it with the appropriate primary antibody overnight. The membranes were washed three times with TBS-0.1% Tween 20 before incubation with secondary antibodies for an hour. The membranes were washed three times with TBS-0.1% Tween 20 and visualized using ECL reagent from Thermo Scientific or Amersham (GE). To detect endogenous ubiquitin, blots were treated and probed as described by Penengo *et al.*^30^.

### Reagents and antibodies

The following antibodies were used for immunoblotting; HA 1:2,000 (Y11, Santa-Cruz biotech), Myc 1:1,000 (9E10, A14, Santa-Cruz biotech), Flag 1:5,000 (Sigma), phospho-SMAD2/3 1:1,000 (S465/467, 138D4, Cell Signaling), SMAD2 1:1,000 (L16D3, Cell Signaling), USP15 1:1,000 (ab56900, Abcam), β-actin 1:10,000 (Sigma), GAPDH 1:10,000 (Santa Cruz), TGF-β receptor I 1:1,000 (R-20, Santa-Cruz biotech), V5 1:2000 (Invitrogen), Ubiquitin 1:1,000 (P4D1, Santa-Cruz biotech). Purified human recombinant TGF-β1 (catalog number: 240-B, R&D Systems) was used for stimulation of cells, it was dissolved in a buffer having trace amounts of BSA and 100 nM Hydrochloric acid. SB431542 (TGF-β inhibitor) was purchased from Selleckchem and dissolved in DMSO.

### Expression plasmids and primers

The expression plasmids pRK-Myc-SMURF2, pCMV-Flag-SMURF2, pCMV-FLAG-USP15, pCMV-Myc-SMURF2 (F29/30A), pRK-HA-ubiquitin or K0-ubiquitin (lysine-less mutant), pCMV-FLAG-SMAD7, pcDNA3-HA-UCH37 were purchased from Addgene. pCMV-FLAG- USP11 was purchased from the MRC unit at the University of Dundee. pCDNA4 USP4 Myc-His was kindly donated by Prof. Peter ten Dijke. V5-USP4 was kindly donated by Prof. Sebastian Nijman. The SMURF2 mutants were made using site-directed mutagenesis kit (Agilent) and confirmed by DNA sequencing. Previously verified short-hairpin sequences against USP4^8^, USP11^10^, USP19^8^ and UCH37^11^ were cloned into pRetroSuper and confirmed by DNA sequencing. pLENTI-shGFP and pLenti-SMURF2 (WT) constructs were kindly provided by Prof. Edward B. Leof (Mayo clinic).

### Generation of CRISPR knockout cell lines

Guide RNA (gRNA) was chosen from the bioinformatically computed genome-wide resource of candidate unique gRNA targets in human exons^31^ and cross referenced with the CRISPR design program for off target effects (crispr.mit.edu). gRNAs with the highest guide percent on target score were cloned into the pX330-U6-Chimeric_BB-CBh-hSpCas9. USP15^1^ gRNA had a target score of 97%. 1 μg of PX330-USP15^1^ vector and 0.1 μg of cmv-GFP were co-transfected into 293T cells. 48 hours after transfection cells were trypsinized and about 1000 cells were plated into 15 cm plates. Single green colonies were picked and expanded and western blot was performed to determine USP15 expression. Clones displaying loss of USP15 expression were further examined. To confirm genomic alterations in the USP15 locus, PCR primers were designed surrounding the USP15^1^ gRNA locus and PCR amplification was performed. PCR products were subsequently transferred into TA cloning vectors and PCR products sequenced.

### Immunoprecipitation

For immunoprecipitation experiments, cells were lysed in ELB buffer (250 mM NaCL, 0.5% Nonidet P-40, 50 mM HEPES, pH 7.3) and supplemented with protease inhibitors. About 500 μg of cell lysates were incubated overnight at 4 °C with the indicated antibodies. The lysates were further incubated with either protein A or protein G sepharose beads (GE Healthcare) for an additional one hour, followed by three washes with the lysis buffer. The beads were boiled in 2x SDS sample buffer and separated on SDS-PAGE gels. When appropriate, cell lysates were immunoprecipitated with anti-FLAG M2 affinity resin (Sigma) for 2 hours at 4 °C and then subsequent steps were followed as mentioned above.

### *In vitro* deubiquitination assay

Flag tagged USP15 was transfected and purified from 293T cells by immunoprecipitation with Flag antibody (Sigma) or with control IgG as described above. Ubiquitinated SMURF2 was obtained by immunoprecipitation with Flag antibody from cellular extracts co-transfected with Flag-SMURF2 and HA-ubiquitin. Immunoprecipitation mixes were then eluted with 450 μg/ml Flag antigen peptide and left on ice for 45 minutes. Ubiquitinated SMURF2 was incubated with purified USP15 or IgG control in 30 μl deubiquitination reaction buffer (500 mM HEPES-KOH [pH 8.0], 150 mM KCL, 5% glycerol, 0.01% Triton X-100, and 2 mM DTT) for 4 hours at room temperature. The entire sample was loaded onto a SDS-Page gel and subjected to immunoblot analysis.

### Sample preparation and protein digestion for Mass spectrometry

Twenty 10 cm dish plates of HEK293T cells were transfected with Flag-SMURF2 (5 μg), K0-Ubiquitin (3 μg), SMAD7 (5 μg) with or without USP15. The cells were lysed with ELB lysis buffer; 30 mg of proteins were immunoprecipitated with Flag antibody resin (Sigma). The beads were incubated for 4 hours at 4 °C and were subjected to competitive elution using flag peptide (Sigma). The eluents were boiled in sample buffer and cast in an SDS-PAGE gel. The gel was then exposed to Coomassie Brilliant Blue staining overnight and then destained. The mono-ubiquitinated and di-mono-ubiquitinated bands of SMURF2 were cut using sterile scalpel and were washed and distained prior to in-gel digestion. Samples were first reduced and alkylated with dithiothreitol and chloroacetamide (Sigma) and was then digested with 5 μg of Trypsin (Promega) overnight in 40 mM NH_4_HCO/10% acetonitrile. Digested samples were sequentially extracted with water/1% formic acid, 50% and 80% acetonitrile. Samples were further desalted with Pierce C-18 Spin Columns (Thermo Scientific), reduced volume to dryness and reconstituted in 2% acetonitrile/0.1% formic acid before mass spectrometry analysis.

### Liquid Chromatography-Mass Spectrometry

NanoLC-MS experiments were conducted on a Dionex UltiMate 3000 RSLC HPLC system coupled to an Orbitrap Q Exactive mass spectrometer equipped with a nanoelectrospray source (Thermo Fisher Scientific). The instruments were controlled by Q Exactive Instrument Software 2.2 SP1 and Dionex Chromatography MS Link software version 2.12. The Orbitrap analyser was mass calibrated with LTQ Velos ESI Positive Ion Calibration Solution at the beginning of the experiment and the spectrometer was set to operate in data-dependent/dynamic exclusion mode to switch automatically between one survey scan followed by up to 20 product ion scans. Survey MS spectra (from *m/z* 350–2000) were acquired with a resolution setting of 70,000 at *m/z* 200 with predictive automatic gain control (pAGC) set to 1E+6. Up to 20 most intense ions with charge states between 2+ and 5+ were sequenced by higher-energy collisional dissociation (HCD) using normalized collision energy of 30%. The ion selection threshold was 5000 counts for HCD and the maximum allowed ion accumulation times were 100 ms for full scans and 100 ms for HCD. Lockmass ion was set to 445.12003 against polysiloxane contamination. Each sample was separated in a New Objective PicoFrit nanospray column (360 μm 75 μm 10 cm) with a 145 minute gradient from 1% to 95% acetonitrile in 0.1% formic acid. The effluent from the HPLC was directly electrosprayed into the mass spectrometer. Spray voltage was set to 2.3 kV. No sheath and auxiliary gas flow was used. Heated capillary temperature was set to 300 °C.

### Data Analysis

Data analysis was performed on Proteome Discoverer version 1.4.0.288 (Thermo Fisher Scientific) using SEQUEST HT sequence database search. Variable modifications were set to Gly-Gly (ubiquitination), phosphorylation and oxidation. The peptide length limit was set to at least 4 amino acids. The confidence of phosphorylation was further analysed with phospho*RS* 3.1 within the Proteome Discoverer platform.

### CAGA Luciferase reporter assay

HEK293T cells were seeded onto a 12 well plate, transfected with CAGA-Luciferase promoter, SV40-Renilla and either an empty vector or the indicated plasmids. The day after transfection, the cells were washed twice with sterile PBS, and replaced with fresh media. 24 hours later, the cells were subjected to an overnight stimulation with or without TGF-β in serum-free media. Thereafter, cells were washed twice with sterile 1x PBS and lysed with 100 μl of Passive Lysis Buffer (Promega). The cells were kept in −80 °C freezer for a minimum of 30 minutes and then thawed at room temperature with gentle shaking. The lysates were analyzed for luciferase and renilla values on a luminometer. Graphs were plotted using Graphpad prism software.

### Lentiviral expression

To produce stable cell lines, HEK293T-FT cells were transfected with pLenti-GFP, SMURF2 (WT), or SMURF2 K734R along with lentiviral packaging constructs (pCMV-VSVG, pMDLg-RRE and pRSV-REV). The media was collected 24 hours later and passed through a 0.45 μm filter, prior to use. MDA-MB-231 cells were infected with the supernatants in the presence of Polybrene (0.01%). The cells were selected and maintained in Puromycin (1.5 μg/10 ml) containing media.

### Wound-healing and transwell migration assays

About 180,000 MDA-MB-231 cells expressing either control vector, SMURF2 (WT) or SMURF2 (K734R) were seeded per well of a six well plate. The cells were serum starved for 24 hours before a scratch was produced, cells were washed with 1x PBS and replenished with 1% serum containing media together with either 5 μM SB431542 or TGF-β (5 ng/ml) for 24 hours. Images were captured immediately after producing the scratch and at 24 hours. For transwell migration assay, MDA-MB-231 cells were grown in 10 cm dishes to 80% confluency and serum-starved for 24 hours, about 50,000 cells were seeded on each transwell migration chamber and treated with either 5 μM SB431542 or 5 ng/ml of TGF-β for 16 hours. The cells were fixed in ice-cold methanol for 10 minutes and stained with crystal violet solution. Migrated cells were then visualized through brightfield microscope and pictures were taken at four random sites and quantified.

### Homology modeling of HECT^SMURF2^: Ub complex

A 3-dimensional structural model of the complex between the HECT domain of SMURF2 protein and donor Ubiquitin (Ub), with the latter covalently attached to the catalytic Cysteine (C716) of SMURF2 was constructed using the crystal structure of a complex between the HECT domain of NEDD4 and Ub (PDB ID: 4BBN)^27^ as template. The sequence identity between the HECT domains of SMURF2 and NEDD4 is ~53% over 380 amino acids overlap which is considered a safe zone for homology modeling^32^. The program MODELLER (version 9.13)^33^ (MODELLER http://salilab.org/modeller/) was used to construct the homology model using the EasyModeller 4.0 graphical interface^34^. The derived model was optimized by subjecting it to energy minimization using steepest descent and conjugated gradient algorithms.

### Molecular model of a ternary HECT^SMURF2^: Ub^1^—Ub^2^ complex

The optimized model of the HECT^SMURF2^: Ub1 complex was used to construct a ternary complex with the secondary ubiquitin (Ub2). The model was created by docking Ub2 and HECT^SMURF2^: Ub using the program HADDOCK through its web portal^35^. The terminal G76 Glycine of Ub2 and K734 Lysine residue from SMURF2 were defined as the active residues for docking, i.e. the docking was carried out under the constraint that G76 and K734 have to be close to each other. A total of 10 structure clusters were generated. The representative structure from the top cluster with the best score as computed by scoring scheme used in HADDOCK was selected as a model for the ternary complex. Subsequently, the structure was optimized by energy minimization using steepest descent and conjugated gradient algorithms.

### System details

The above models of binary and ternary complex were next subject to detailed molecular dynamics simulations. A thioester bond was constructed between the terminal G76 Glycine of the donor ubiquitin (Ub1) and the catalytic C716 Cysteine of SMURF2 (backbone carbonyl carbon “C” – side chain sulphur “SG”). An isopeptide bond was constructed between the terminal G76 Glycine of the secondary ubiquitin (Ub2) and K734 Lysine residue of SMURF2 (backbone carbonyl carbon “C” – side chain nitrogen “NZ”). Both bonds were constructed using the XLEAP module of the modeling and simulations program AMBER12. RESP (Restrained Electrostatic Potential) based atomic charges for the modified amino acids involved in the aforementioned bonds were derived through the R.E.D. server using RESP-A1A (HF/6-31G*) charge model and Guassian_2009_C.01 quantum mechanics program^36^. Other force field parameters were obtained from the all-atom ff99SB^37^ force field of AMBER12. The N- and C-terminals of the HECT^SMURF2^ domain were capped with ACE and NME moieties respectively to keep them neutral. The structures were placed in a cuboid box filled with water molecules that were modeled with the TIP3P potential^38^ such that the minimum distance from the edge of the box was 10 Å. The net charge of the binary complex was −6 and that for the ternary complex was −7 which was accordingly neutralized by adding six and seven sodium ions respectively using the TLEAP module of AMBER12.

### Molecular dynamics simulations

Molecular Dynamics (MD) simulations were carried out in AMBER12 software package using the PMEMD module with the all-atom ff99SB^36^ force field parameters. The solvated and energy minimized complexes were heated to a temperature of 300 K, equilibrated for 500 ps and finally subjected to 100 ns of simulations. The NVT ensemble was used for heating followed by NPT conditions thereafter. Temperature was regulated using Langevin dynamics^39^,^40^ with a collision frequency of 1.0 ps^−1^ and pressure was maintained at 1 atm using a weak-coupling^41^ with a relaxation time of 1 ps. Periodic boundary conditions were applied and the Particle Mesh Ewald^42^ method (PME) was used for calculating long range electrostatic interactions. All bonds involving hydrogen atoms were constrained using the SHAKE algorithm^43^ with an integration time-step of 2 fs. The atomic coordinates were saved every 25 ps. The generated ensemble of structures was clustered using the average-linkage algorithm^44^. Hydrogen-bond analysis was done using the PTRAJ module in AMBER12. The graphical representations of the molecular models were created using the PyMOL molecular visualization software.

### Statistical analyses

Students’s *t* tests were performed for statistical analyses. Data in all graphs represent mean S.D.

## Additional Information

**How to cite this article**: Iyengar, P. V. *et al.* USP15 regulates SMURF2 kinetics through C-lobe mediated deubiquitination. *Sci. Rep.* **5**, 14733; doi: 10.1038/srep14733 (2015).

## Supplementary Material

Supplementary Information

Supplementary Movie S1

Supplementary Movie S2
