# Supplementary material for: USP15 regulates SMURF2 kinetics through C-lobe mediated deubiquitination
Source: Sci Rep. 2015 Oct 5;5:14733. doi: 10.1038/srep14733 (PMC4593006; doi:10.1038/srep14733)
Supplement: Supplementary Information [file srep14733-s1.doc]

**USP15 regulates SMURF2 kinetics through C-lobe mediated deubiquitination**

Prasanna Vasudevan Iyengar, Patrick Jaynes, Laura Rodon, Dilraj Lama, Kai Pong Law, Yoon Pin Lim, Chandra Verma, Joan Seoane, Pieter Johan Adam Eichhorn

**Supplementary Figure S1**

**
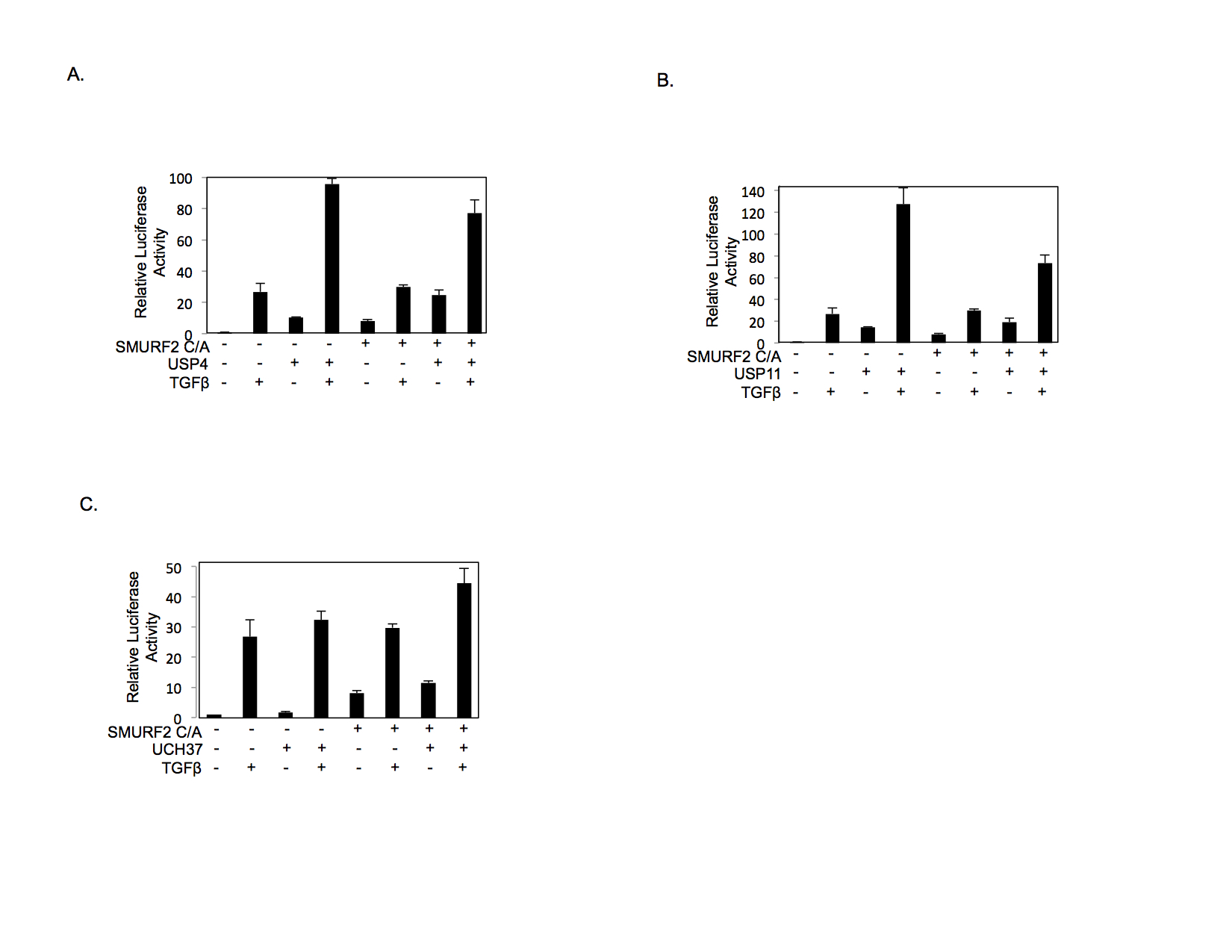
**

**Supplementary Figure S2**

**
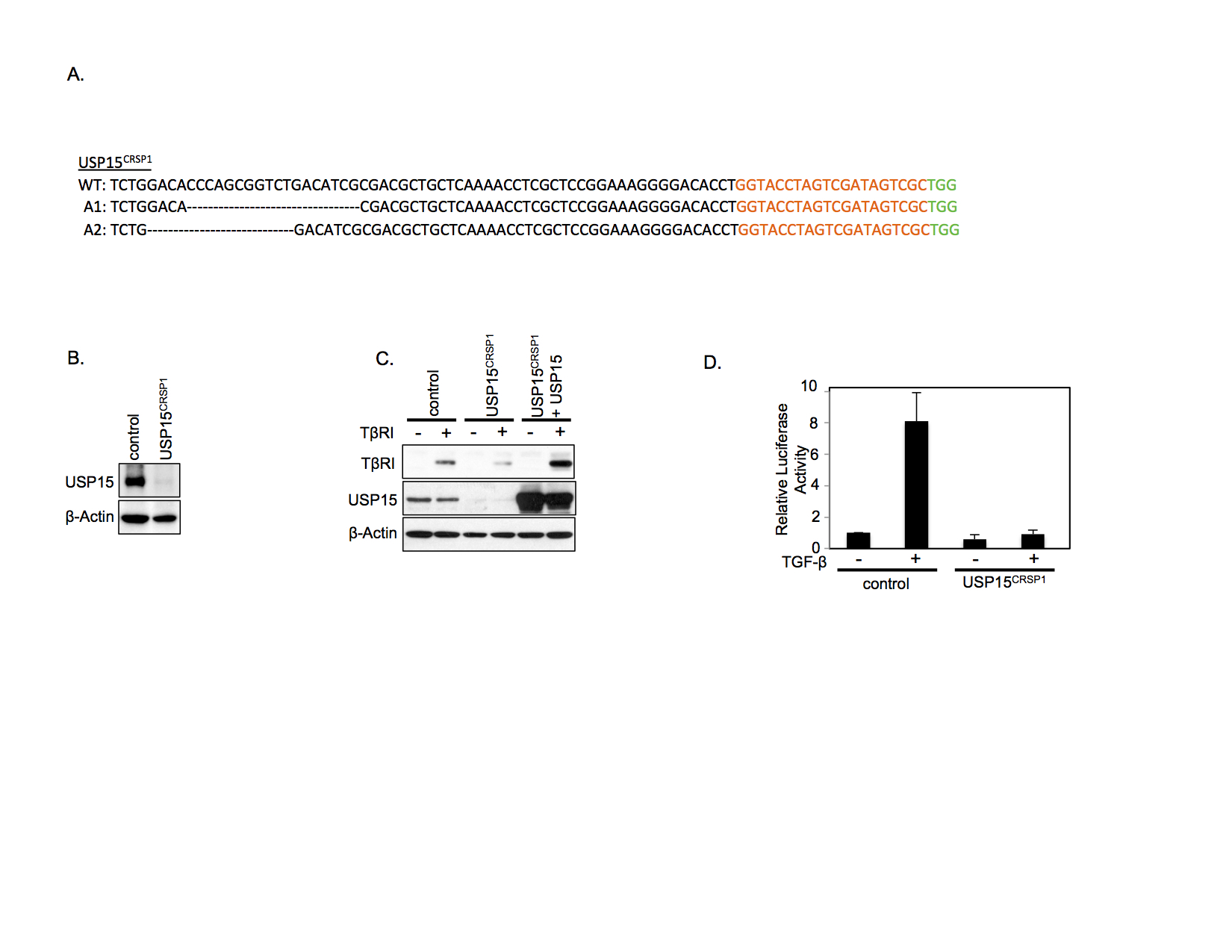
**

**Supplementary Figure S3**

**
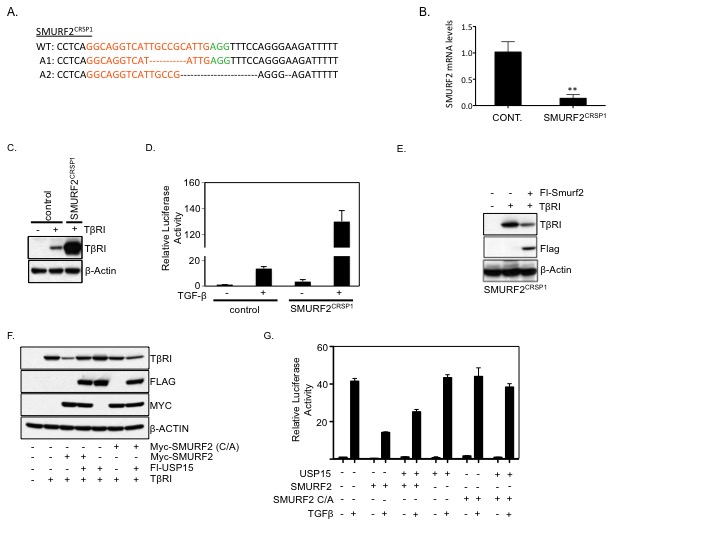
**

**Supplementary Figure S4**

**
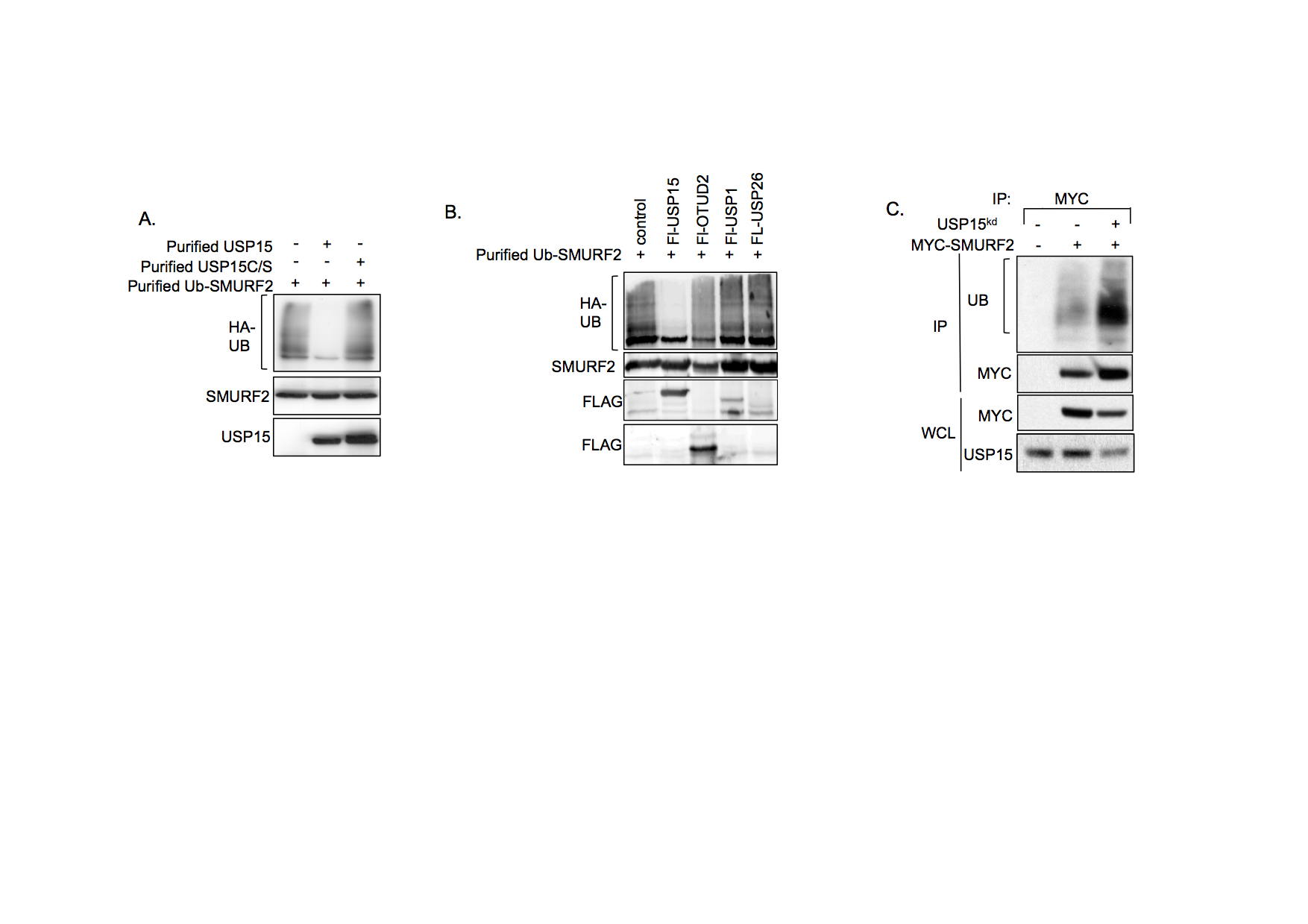
**

**Supplementary Figure S5**

**
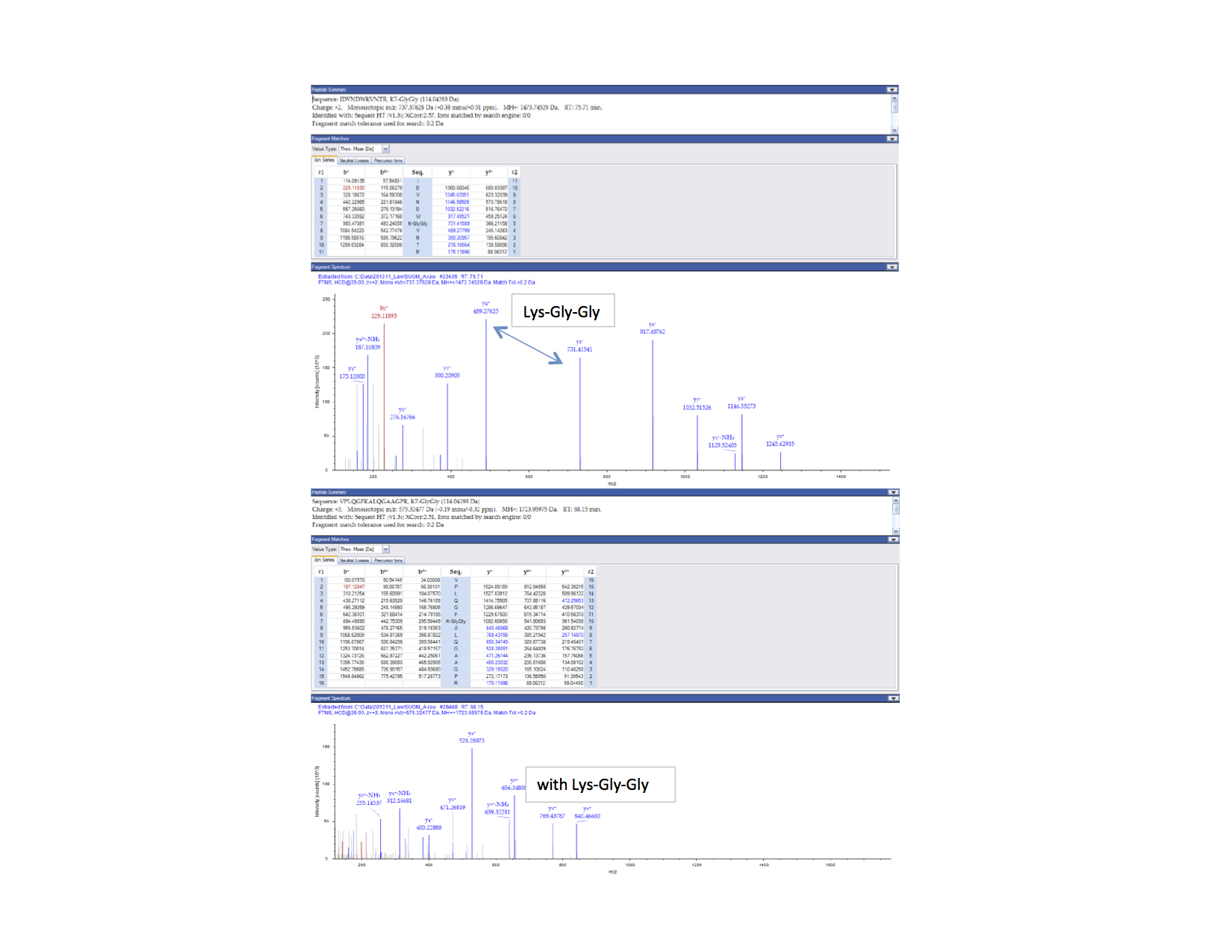
**

**Supplementary Figure S6**

**
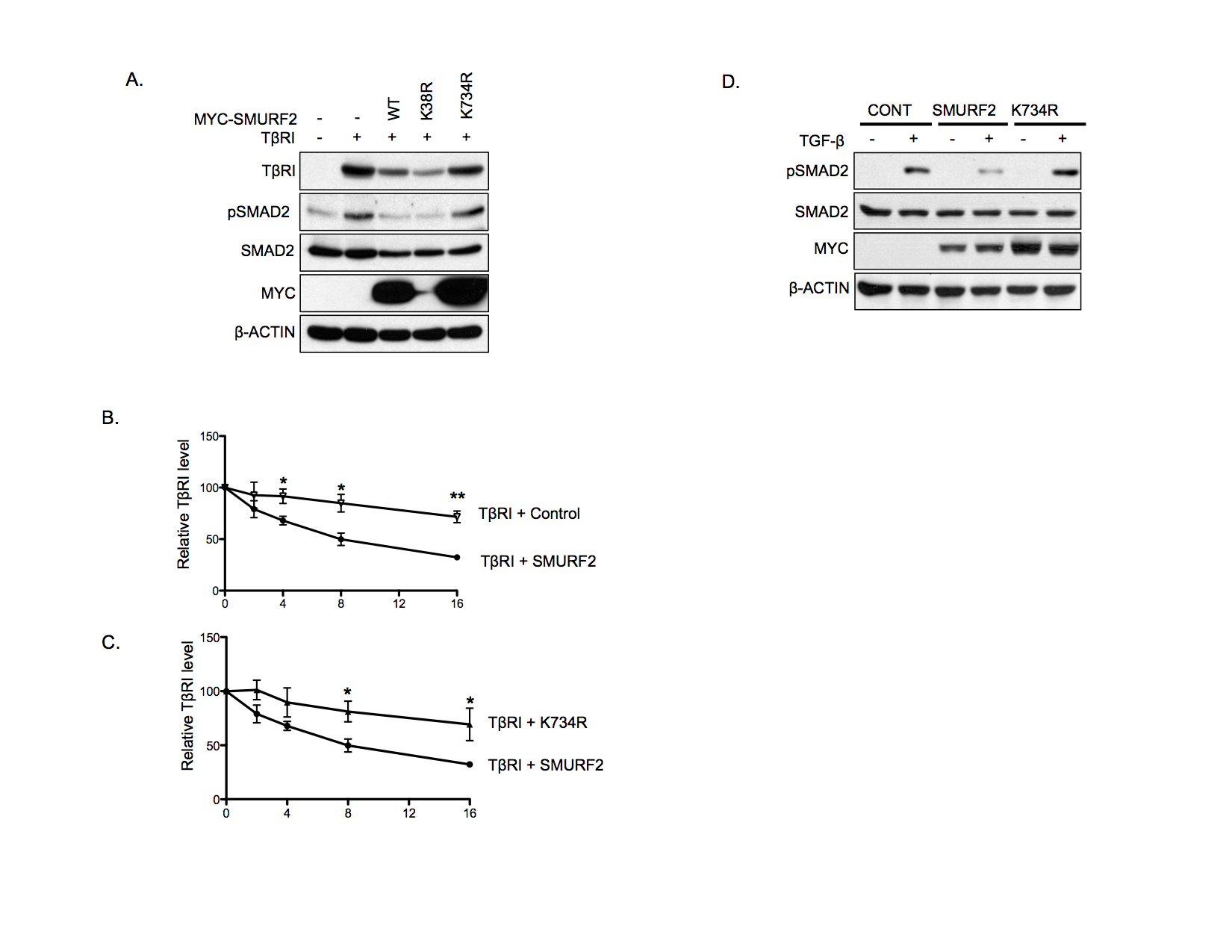
**

**Supplementary Figure S7**

**
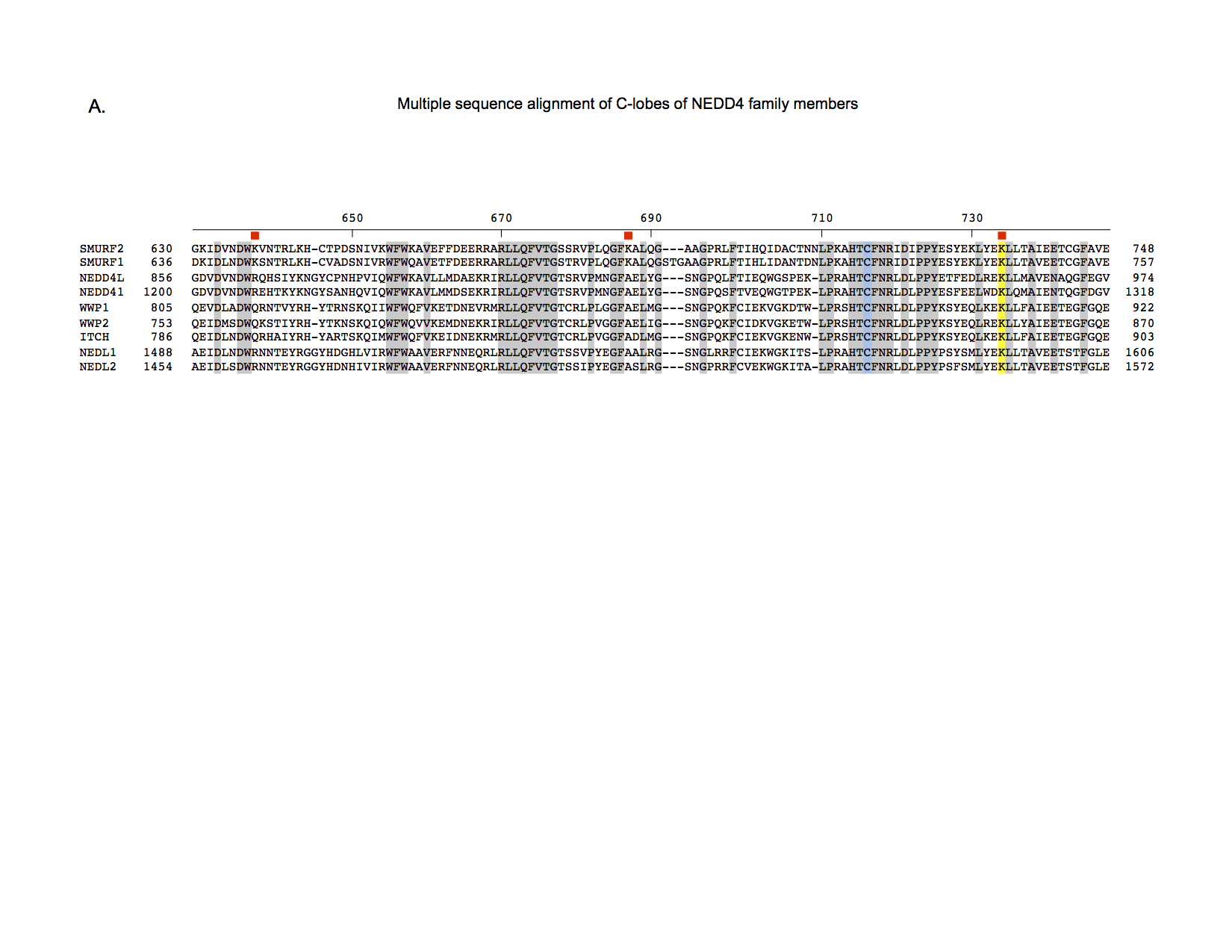
**

**Supplementary Figure S8**

**
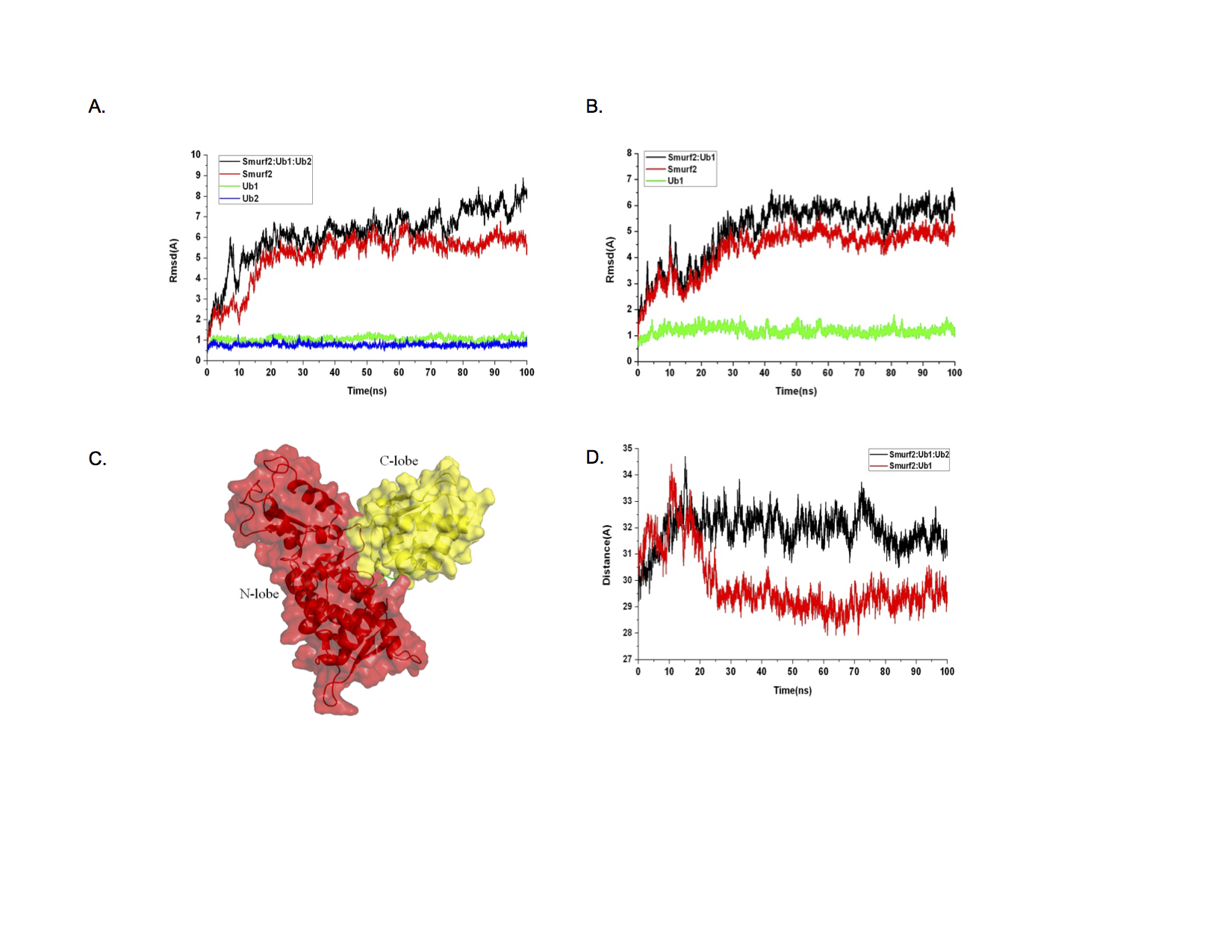
**

**Supplementary Figure S9**

**
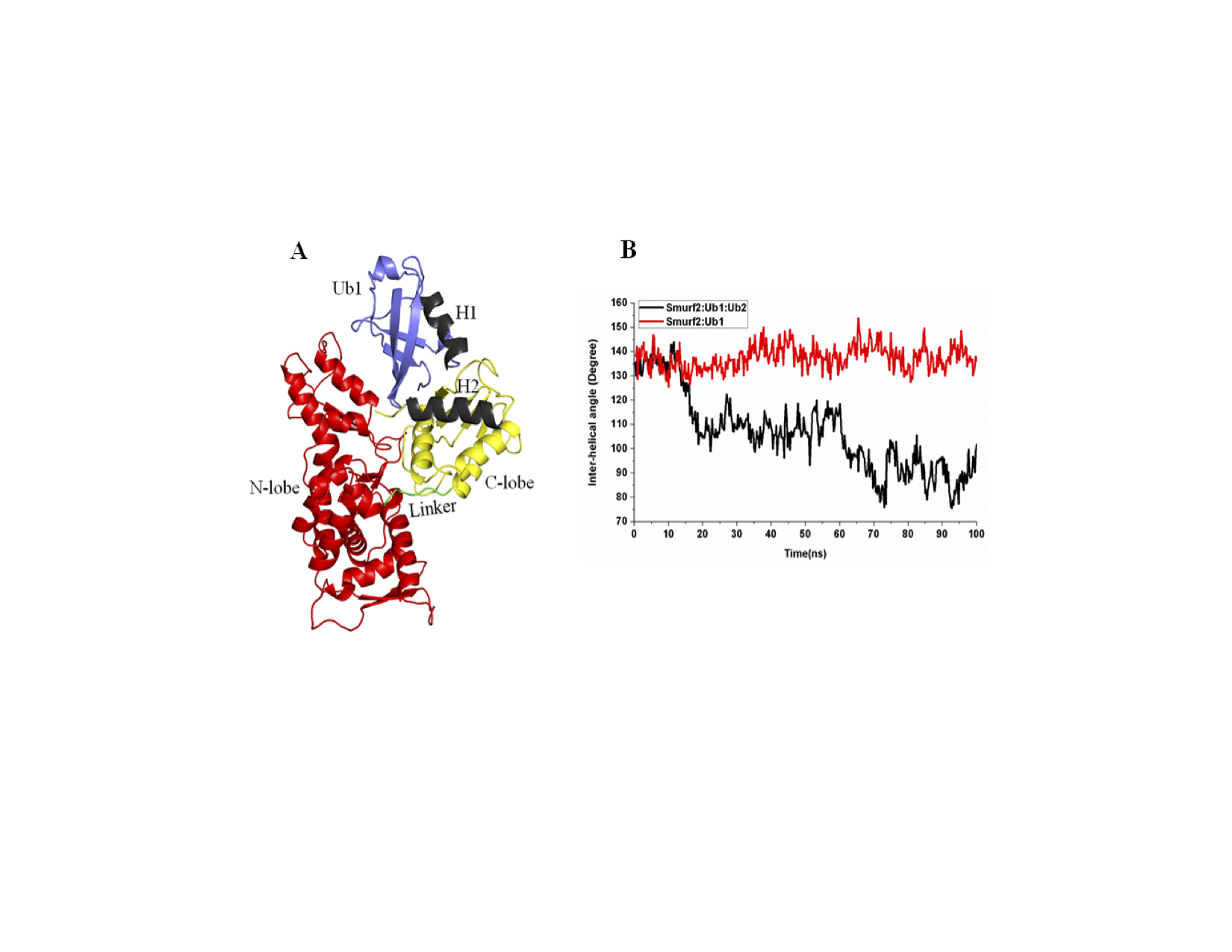
**

**Supplementary Figure S10**

**Table 1**

| **Residue pairs Common to both**  **Smurf2: Ub1:Ub2**  **and**  **Smurf2:Ub1** | **Residue pairs present only in**  **Smurf2:Ub1** | **Residue pairs present only in**  **Smurf2:Ub1:Ub2** |
| --- | --- | --- |
| A713-R74, H714-G75, **A713-G75 (O-N)**, **N709-Q40 (OD1/ND2-NE2/OE1),** K712-L73, **T715-G75 (N-O)**, N709-P37, P711-L73, F674-L73, F674-R74, H714-G76, **E741-T9(OE1/OE2-OG1),** L710-L73, T742-L73, K712-Q40, T715-G76, C716-G76, A738-L73, Q673-R74, L710-I36, C743-R74, T715-R74 | C716-G76, **K712-Q40 (NZ-OE1)**, C716-G75, N709-I36, E741-T7, E741-L8, V534-R72, T715-R74, N709-G35, T742-R74, **N708-K33(ND2-O)**, N708-E34, E740-T9, N708-G35, L710-L71, E538-Q49, F745-L8, E741-L71, E741-L69 | N708-Q31, G677-R74, L710-Q40, K712-R72, **E741-L8(OE2-N)**, T742-L71, N708-P37, N709-D39, K712-D39, C743-L73 |

**Supplementary Figure legends**

**Supplementary Figure 1**

(**A**) Luciferase assay representing TGF-β activity in 293T cells transfected with either catalytically inactive mutant of SMURF2 (C/A) and USP4, USP11 (**B**), or UCH37 (**C**). Cells were treated overnight with TGF-β (2.5 ng/ml). Data are mean ± s.d.

**Supplementary Figure 2**

(**A**) 293T cells transfected with pX330-U6-Chimeric_BB-CBh-hSpCas9 vector containing guide RNA targeting USP15 display a frameshift mutations in both alleles (A1, A2) of genomic USP15. Guide RNA sequence is denoted in red and PAM sequence is denoted in green. (**B**) Immunoblot analysis of 293T cells and USP15^CRSP1^ cells showing knockout of USP15 in CRISPR knockout cells. (**C**) Immunoblot analysis of 293T cells or USP15^CRSP1^ cells transfected with TβRI with or without reconstituted USP15 expression. Whole cell lysates were probed with the indicated antibodies. (**D**) Luciferase assay in 293T and USP15^CRSP1^ cells transfected with CAGA-luciferase reporter and treated overnight with TGF-β (2.5 ng/ml). Data are mean ± s.d.

**Supplementary Figure 3**

(**A**) 293T cells transfected with pX330-U6-Chimeric_BB-CBh-hSpCas9 vector containing guide RNA targeting SMURF2 display a frameshift mutations in both alleles (A1, A2) of genomic SMURF2. Guide RNA sequence is denoted in red and PAM sequence is denoted in green. (**B**) Quantitative RT-PCR (qRT-PCR) of SMURF2 in HEK293T cells or SMURF2^CRSP1^ cells. *GAPDH* mRNA levels were used as an internal normalization control. ** *P* value = 0.002 using Student’s *t* test. Data are mean ± s.d. (**C**) Immunoblot analysis of 293T cells or SMURF2^CRSP1^ cells transfected with TβRI. Whole cell lysates were probed with the indicated antibodies. (**D**) Luciferase assay in 293T and SMURF2^CRSP1^ cells transfected with CAGA-luciferase reporter and treated overnight with TGF-β (2.5 ng/ml). Data are mean ± s.d. (**E**) Immunoblot analysis of SMURF2^CRSP1^ cells transfected with TβRI with or without reconstituted SMURF2 expression. Whole cell lysates were probed with the indicated antibodies. (**F**) Immunoblot analysis of SMURF2^CRSP1^ cells transfected with TβRI with or without reconstituted SMURF2 or SMURF2 C/A in combination with USP15. (**G**) Luciferase assay in SMURF2^CRSP1^ cells transfected with CAGA-luciferase reporter and SMURF2 or SMURF2 C/A, USP15, or both and treated overnight with TGF-β (2.5 ng/ml). Data are mean ± s.d.

**Supplementary Figure 4**

(**A**) An *in vitro* deubiquitination experiment using ubiquitinated SMURF2 purified from transfected 293T cells with either purified USP15 or its catalytically inactive form (USP15C/S). The samples were immunobloted with the indicated antibodies. (**b)** An *in vitro* deubiquitination assay using ubiquitinated SMURF2 purified from transfected 293T cells and the indicated purified deubiquitinating enzymes. The samples were immunobloted with the indicated antibodies. (**C**) Immunoprecipitation with anti-Myc antibody in 293T cells transfected with Myc-SMURF2 and shRNA targeting USP15; an immunoblot analyses of indicated proteins is shown.

**Supplementary Figure 5**

MS/MS spectrum of the C-Lobe peptide of SMURF2 K638 (top panel) or K687 (bottom panel) with the diglycine modified lysine residue. Peptide sequence is shown indicating the change in molecular weight pertaining to Ub size.

**Supplementary Figure 6**

(**A**) Immunoblot analysis in 293T cells expressing TβRI in the presence of either wild type SMURF2 or SMURF2 K38 or SMURF2 K734. Whole cell lysates were probed with the indicated antibodies. (**B**) Quantification of the band intensities of TβRI from Figure 4D; results are shown as SEM of three independent experiments. **P*<0.05, ***P*<0.01 using Student’s *t* test. (**C**) Quantification of the band intensities of TβRI from Figure 4D; results are shown as SEM of three independent experiments. **P*<0.05 using Student’s *t* test. (**D**) Immunoblot analysis of 293T cells transfected with SMURF2 or SMURF2 K734 and treated overnight with TGF-β (2.5 ng/ml). Whole cell lysates were probed with the indicated antibodies.

**Supplementary Figure 7**

(A) C-lobe sequences of NEDD4 family E3 ligases were aligned using CLUSTALW 2.1; conserved residues are highlighted in grey, catalytic cysteine in blue and conserved lysine in yellow (K734 of SMURF2). Ubiquitinated sites on the C-lobes have been indicated with a red square above the alignment. Amino acid numbering for SMURF2 is indicated above the alignment.

**Supplementary Figure 8**

RMSD (Backbone heavy atoms) of **(A)** SMURF2:Ub1:Ub2 and **(B)** SMURF2:Ub1 system with reference to the initial structure of the simulation. The global change in RMSD by the end of the simulation period is ~6Å for the SMURF2:Ub1:Ub2 complex and ~5.5Å for the SMURF2:Ub1 complex. The deviation in the modeled structure is largely contributed by the SMURF2 protein as can be seen from the RMSD of the individual subunits in both the complexes. **(C)** Schematic of SMURF2 protein denoted by N-lobe (red) and C-lobe (yellow) domains of SMURF2 connected by a linker (green). **(D)** Time evolution of center of mass distance between the N- and C-lobe domains of SMURF2. This correlates nicely with RMSD profile and contributes significantly towards the higher deviation observed from the analysis.

**Supplementary Figure 9**

**(A)** SMURF2:Ub1 complex indicating the location of Helices H1 and H2 (black colors) in Ub1 and C-lobe domain of SMURF2 respectively. **(B)** Inter-helical angle between H1 and H2 during the simulation. The angle was calculated using interhlx (K.Yap, University of Toronto).

**Supplementary Figure 10**

As part of a negative feedback loop SMAD7 recruits SMURF2 to the TGF-β receptor complex to attenuate TGF-β signaling. USP15 regulates TGF-β receptor stability by deubiquitinating both the E3 ligase SMURF2 and its substrate the TGF-β receptor complex to protect TGF-β output. USP15 may also play a role in regulating SMAD3 ubiquitination, a known substrate for both SMURF2 and USP15, in a similar manner.

**Table S1**

**Residue-wise interaction between SMURF2 and Ub1:** The analysis was done for the last 50 ns of the simulation period. The cut-off distance for residue contact was 4.5 Å between at least a pair of heavy atoms. The hydrogen-bond analysis was done with distance cut-off of 3.5Å and angle 120°. Only those residue-pairs are reported which satisfy the criteria in at least 50% of the analyzed structures. The residue pairs which are involved in hydrogen-bond interaction are highlighted in bold and underlined with the specific atoms involved in the bond shown in brackets.

**Supplementary Movie S1**: MD simulated trajectory of SMURF2:Ub1 complex. The N-lobe and C-lobe domains of SMURF2 protein are shown in red and blue colours respectively. Ubiquitin Ub1 is shown in blue color. The representative helices H1 and H2 from Ub1 and C-lobe respectively are coloured in black. The trajectory highlights the domain motion of N- and C-lobe domains and the orientation of Ub1 with respect to C-lobe domain.

**Supplementary Movie S2**: MD simulated trajectory of SMURF2:Ub1:Ub2 complex. The N-lobe and C-lobe domains of SMURF2 protein are shown in red and blue colours respectively. Ubiquitin Ub1 and Ub2 is shown in blue and green colours. The representative helices H1 and H2 from Ub1 and C-lobe respectively are coloured in black. The trajectory highlights the domain motion of N- and C-lobe domains and the reorientation of Ub1 in the presence of Ub2.
